# How COVID-19 affected academic publishing: a 3-year study of 17 million research papers

**DOI:** 10.1093/ije/dyaf058

**Published:** 2025-05-27

**Authors:** Matthew Whitaker, Sabrina Rodrigues, Graham Cooke, Bérangère Virlon, Christl A Donnelly, Helen Ward, Paul Elliott, Marc Chadeau-Hyam

**Affiliations:** School of Public Health, Imperial College London, London, UK; MRC Centre for Environment and Health, Imperial College London, London, UK; School of Public Health, Imperial College London, London, UK; MRC Centre for Environment and Health, Imperial College London, London, UK; Imperial College Healthcare NHS Trust, London, UK; Department of Infectious Disease, Imperial College London, London, UK; National Institute for Health Research Imperial Biomedical Research Centre, London, UK; Institut Pasteur, Université Paris Cité, Paris, France; School of Public Health, Imperial College London, London, UK; Department of Statistics, University of Oxford, St Giles’, Oxford, UK; Pandemic Sciences Institute, University of Oxford, Oxford, UK; School of Public Health, Imperial College London, London, UK; Imperial College Healthcare NHS Trust, London, UK; National Institute for Health Research Imperial Biomedical Research Centre, London, UK; MRC Centre for Global Infectious Disease Analysis and Jameel Institute, Imperial College London, London, UK; School of Public Health, Imperial College London, London, UK; MRC Centre for Environment and Health, Imperial College London, London, UK; Imperial College Healthcare NHS Trust, London, UK; National Institute for Health Research Imperial Biomedical Research Centre, London, UK; Health Data Research (HDR) UK London at Imperial College, London, UK; UK Dementia Research Institute at Imperial College, London, UK; School of Public Health, Imperial College London, London, UK; MRC Centre for Environment and Health, Imperial College London, London, UK

**Keywords:** COVID-19, bibliometrics, pandemics, Altmetrics, policy

## Abstract

**Background:**

The COVID-19 pandemic induced an unprecedented response from the scientific research community. Previous studies have described disruption of the norms of academic publishing during this time. This study uses an epidemiological statistical toolkit alongside machine-learning methods to investigate the functioning of the scientific information-generation and -consumption ecosystem throughout the pandemic.

**Methods:**

A dataset of 17 million scientific research papers that were published between January 2019 and December 2022 was analysed. Data on citations and Altmetrics were harvested, and topic modelling was applied to abstracts. COVID-19-related articles were identified from title text. We investigated publication dynamics, correlations between citation metrics and Altmetrics, rates of publication in preprints, and temporal trends in topics, and compared these metrics in COVID-19 papers vs non-COVID-19 papers.

**Results:**

Throughout 2020–2, 3.7% of English-language research output was on the topic of COVID-19. Journal articles on COVID-19 were published at a consistent rate during this period, while preprints peaked in early 2020 and decreased thereafter. COVID-19 preprints had lower publication rates in the peer-reviewed literature than other preprints, particularly those that were preprinted during early 2020. COVID-19 research received significantly more media and social media attention than non-COVID-19 research, and preprints received more attention, on average, than journal articles, with attention peaking during the initial wave and subsequent peaks corresponding to the emergence of novel variants. COVID-19 articles exhibited a higher correlation between Altmetrics and citation metrics compared with non-COVID-19 publications, suggesting a strong alignment between scientific and public attention.

**Conclusion:**

This study provides a comprehensive description of the rapid expansion of COVID-19 research, revealing evolving research areas and waxing and waning public interest across different topics. Preprints played an important role in disseminating scientific findings, but the level of coverage of preprinted findings emphasizes the need for guidelines in handling preprint research in media, particularly during a pandemic.

Key Messages

- A well-functioning information ecosystem is vital in a public health crisis; this study investigates how well the scientific information-generation and -consumption ecosystem worked under the acute stress of the COVID-19 pandemic.
- We describe a large wave of COVID-19-related publications and preprints, and a corresponding spike in academic and public consumption of these papers; COVID-19 preprints received more public attention than peer-reviewed papers, on average, and were less likely to be published in peer-reviewed journals than non-COVID-19 preprints.
- The findings shed light on the functioning of the quality-control mechanisms of science under the stress of a public health crisis and underline the need for guidelines on public and media engagement with non-peer-reviewed research.

## Introduction

Effective public health measures rely on the efficient creation, validation, dissemination, and interpretation of research. The COVID-19 pandemic has induced an unprecedented response from the scientific research community, but this has presented a stress test for the institutions and incentive structures of scientific publishing and communication.

One effect has been the strain on peer-review systems [1–3]. Schonhaut *et al.* [4] found that, while peer-review times were faster, COVID-19 withdrawal rates far exceeded those of influenza (0.26% vs 0.023%). Yeo-Teh *et al.* also found a high retraction rate for COVID-19 papers [5].

Demand for rapid publication led to a substantial surge in preprint usage: medRxiv submissions increased 10-fold between January and May 2020 [6]. Preprints were also widely reported and shared: medRxiv received >10 million monthly page views at the pandemic peak [6], with significant implications for public health messaging [7].

Beyond quality-control concerns in COVID-19 papers, previous research has described a rapid redirection of research efforts into COVID-19, with potential adverse effects on scientific output in non-COVID-19 fields, including fewer new projects and less time available for other research [8–10].

Here, we use the tools of epidemiology, bibliometrics, and machine learning to describe and analyse how the scientific publishing ecosystem functioned under the acute stress of a pandemic. Using a large corpus of all research literature (>17 million papers) published between the start of 2019 and the end of 2022, we provide a descriptive analysis of all published COVID-19 research literature and investigate questions relating to the functioning of the information-generation and -consumption parts of the scientific ecosystem.

## Methods

### Dataset

We extracted data on published research papers and preprints from 1 January 2019 to 31 December 2022 from the Dimensions database [11].

Articles were classified as COVID-19-related if the title text returned a positive result for a Boolean search for COVID-19-related words (Supplementary Methods S1.2).

In addition to the Dimensions extract, we harvested additional data points for each paper. The citation-based metrics of scientific attention and scientific visibility were citation count (at the time of data export, March 2023); citation rate (citation count/days since publication); field citation ratio (citations normalized to the field of research and publication date of the paper); journal Impact Factor (IF—the average citations received by the article of that journal within a 2-year window); and Journal Citation Ratio (JCR—a field-normalized version of the IF). As a metric of public attention, we used the Altmetrics score: a weighted sum of attention across tracked digital platforms. Altmetrics data were gathered through the Altmetrics API by using the R package rAltmetric [12, 17]. Journal-level impact and citation data were gathered from the Thomson Reuters journal citation reports service [13]. Where preprints were later published in a journal, the preprint was linked to the publication.

### Descriptive analyses

Primary descriptive analyses were conducted to characterize the dataset, including summary tables and plots of published COVID-19 literature. Correlation analysis was conducted to examine the relationships between the metrics of academic article impact (citation metrics and publishing journal impact metrics) and non-academic article impact (Altmetrics), and to see how these relationships vary between COVID-19 and non-COVID-19 papers, and between journal articles and preprints. Correlations were calculated with Spearman’s rank correlation, by using pairwise complete cases, and visualized by using heat maps.

To investigate the changing rates of publishing output, linear regression models were fit to the monthly publication data. To test whether the pandemic era was associated with a change in the rate of publication of non-COVID-19 research, a further regression model was fitted to the monthly publication data including a binary independent variable denoting the pre-pandemic (2019) or pandemic era (2020–2), including an interaction between the independent variables (Supplementary Methods S1.4).

### Rates of subsequent publication in preprints

The rates of publication within 1 year and 2 years were calculated for preprints. Additionally, time-to-event analysis was conducted on preprints from 2019 to 2022. The ‘event’ was publication. Cox proportional-hazards models were fitted with *y* as the time-to-publication and *X* as a categorical variable indicating the category of the preprint: either (i) pre-pandemic, (ii) non-COVID-19, or (iii) COVID-19. Time-to-event data were also visualized as Kaplan–Meier plots. As sensitivity analyses, we repeated the analyses on preprints published between 1 January 2020 and 1 August 2020, and on preprints from medRxiv and bioRxiv only.

### Topic modelling

To infer underlying thematic structure, topic modelling was applied to COVID-19 paper abstracts using BERTopic [14]. For a full description of the text preprocessing and BERTopic modelling process, see Supplementary Methods (section S1.5).

To investigate the relationship between article topics and (i) citation rates and (ii) media attention, univariable linear regression models were fitted, with the response being either the citation rate or the Altmetrics score and the predictor as the topic probability value—a measure of the association between a document and a topic. All topics were modelled like this for each outcome and results were visualized as volcano plots. Estimated regression coefficients were compared across outcomes to see whether the same associations existed for citations as for Altmetrics. To examine the evolving drivers of attention over time, this analysis was repeated in rolling 8-week time windows (wide enough to smooth out individual-paper effects but narrow enough to capture short-term fluctuations) throughout 2020–2 (Supplementary Methods S1.6).

## Results

### Data overview

**Figure 1.**
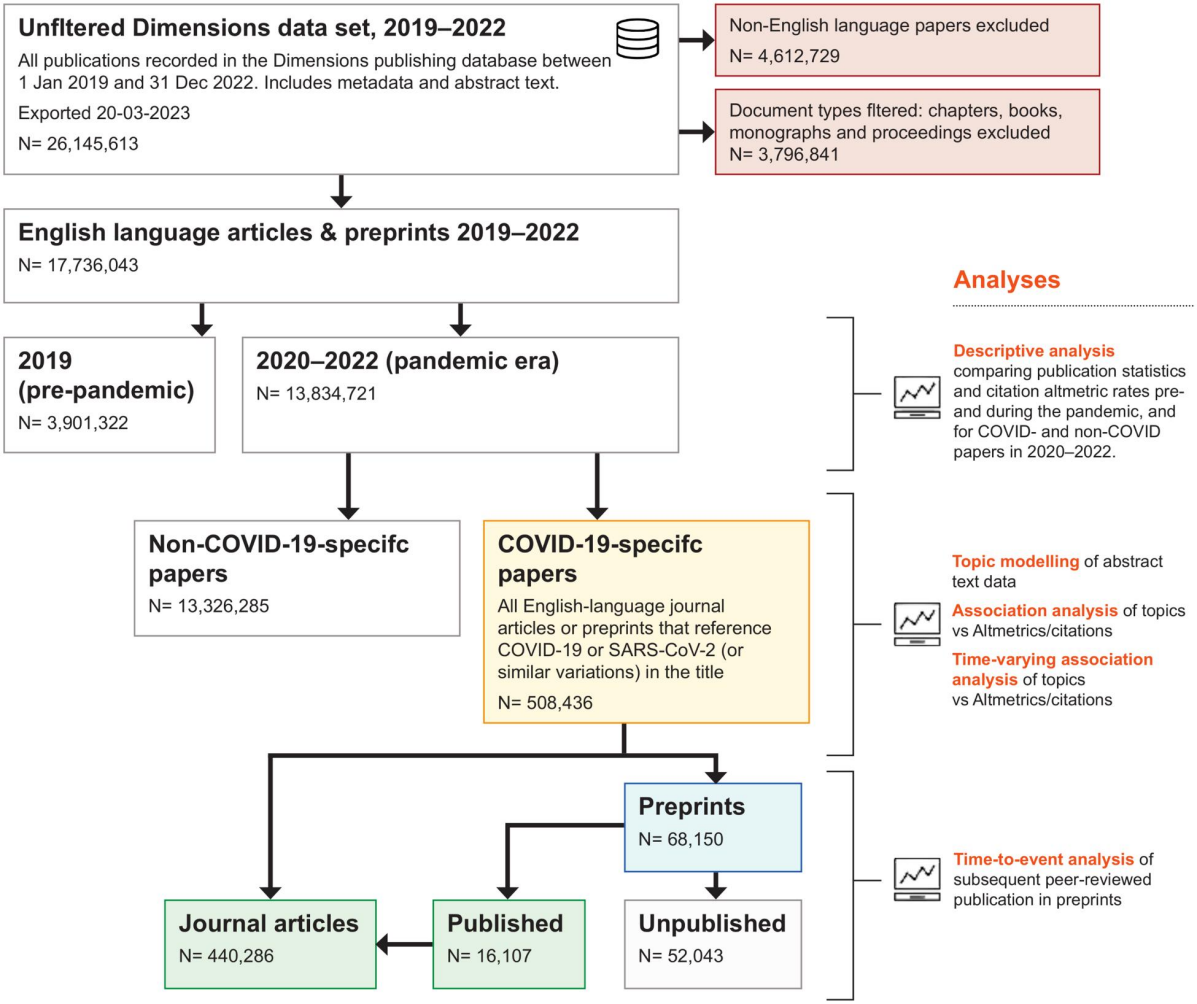
Flow chart showing study corpus and exclusions.

After exclusions (Figure 1), the final dataset comprised 17 736 043 papers, of which 508 436 were classified as COVID-19 papers (440 286 journal articles, 68 150 preprints). This constituted 3.5% of the 12 538 073 English-language journal articles and 5.3% of the 1 296 648 preprints published during 2020–2 (Figure 1 and Table 1).

**Table 1.** Summary of all papers indexed in the Dimensions database in 2019, 2020, 2021, and 2022. Papers are separated into preprints and journal articles, and into COVID-19 and non-COVID-19 papers. COVID-19 papers are so designated if COVID-19/SARS-CoV-2 (or similar variations) appear in the article title

		2019		2020–2				2019–22
				Non-COVID-19		COVID-19		
Variable	Level	Preprint	Journal article	Preprint	Journal article	Preprint	Journal article	Overall
*N*		263 371 (100%)	3 637 951 (100%)	1 228 498 (100%)	12 097 787 (100%)	68 150 (100%)	440 286 (100%)	17 736 043 (100%)
Year	2019	263 371 (100%)	3 637 951 (100%)	0 (0%)	0 (0%)	0 (0%)	0 (0%)	3 901 322 (22%)
	2020	0 (0%)	0 (0%)	368 782 (30%)	3 900 826 (32.2%)	32 183 (47.2%)	118 244 (26.9%)	4 420 035 (24.9%)
	2021	0 (0%)	0 (0%)	395 076 (32.2%)	4 092 453 (33.8%)	22 088 (32.4%)	171 298 (38.9%)	4 680 915 (26.4%)
	2022	0 (0%)	0 (0%)	464 640 (37.8%)	4 104 508 (33.9%)	13 879 (20.4%)	150 744 (34.2%)	4 733 771 (26.7%)
Published in journal	No	155 671 (59.1%)	3 637 951 (100%)	897 517 (73.1%)	12 097 787 (100%)	52 043 (76.4%)	440 286 (100%)	17 281 255 (97.4%)
	Yes	107 700 (40.9%)	–	330 981 (26.9%)	–	16 107 (23.6%)	–	454 788 (2.6%)
Published in journal within one year	No	178 681 (67.8%)	3 637 951 (100%)	951 336 (77.4%)	12 097 787 (100%)	53 177 (78%)	440 286 (100%)	17 359 218 (97.9%)
	Yes	84 690 (32.2%)	–	277 162 (22.6%)	–	14 973 (22%)	–	376 825 (2.1%)
Published in journal within 2 years	No	167 036 (63.4%)	3 637 951 (100%)	931 181 (75.8%)	12 097 787 (100%)	52 445 (77%)	440 286 (100%)	17 326 686 (97.7%)
	Yes	96 335 (36.6%)	–	297 317 (24.2%)	–	15 705 (23%)	–	409 357 (2.3%)
Number of citations	Mean (SD)	0.58 (3.46)	9.87 (31.23)	0.3 (2.3)	4.05 (18.95)	5.3 (22.94)	16 (131.76)	5.23 (29.81)
		0 (0)	3 (11)	0 (0)	1 (4)		2 (9)	1 (4)
Relative citation ratio	Mean (SD)	–	1.38 (3.16)	0.89 (1.95)	1.55 (6.47)	1.65 (3.09)	4.6 (21.32)	1.64 (7.17)
	Median (inter-quartile range (IQR))	–	0.8 (1.22)	0.39 (0.7)	0.92 (1.33)		1.65 (3.33)	0.9 (1.35)
Field citation ratio	Mean (SD)	0.24 (1.55)	3.42 (10.46)	0.24 (1.9)	2.86 (11.95)	3.35 (12.21)	13.53 (75.57)	3.02 (15.72)
	Median (IQR)	0 (0)	1.24 (3.71)	0 (0)	1.01 (3.19)		3.28 (9.93)	0.85 (3.09)
Altmetrics score	Mean (SD)	5.49 (42.23)	4.29 (44.07)	4.08 (35.38)	3.88 (46.06)	45.74 (365.19)	38.31 (383.52)	5.02 (78.47)
	Median (IQR)	1 (3)	0 (1)	1 (2)	0 (1)		1 (5)	0 (1)
Altmetrics: citations ratio (logged)	Mean (SD)	4.04 (2.95)	1.87 (2.93)	3.56 (2.79)	1.86 (2.87)	4.19 (3.56)	3.16 (3.28)	2.06 (2.94)
Time from preprint to article publication	Mean (SD)	194.93 (194.72)	–	140.43 (158.27)	–	141.74 (121.46)	–	153.52 (168.15)

### Dynamics of scientific production

**Figure 2.**
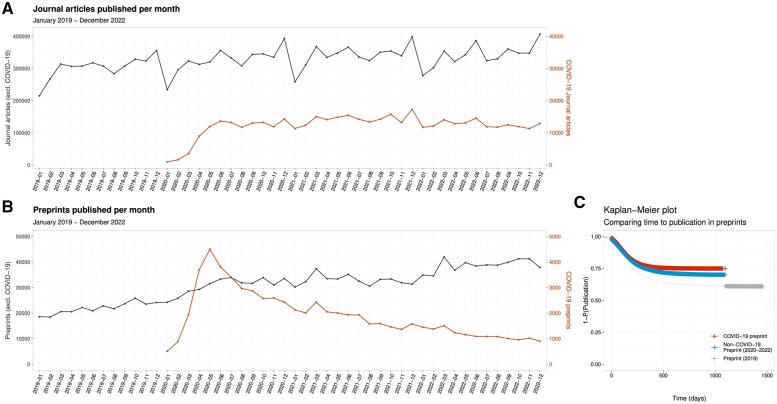
(A) Comparison of monthly publication numbers of journal articles and (B) preprints between 2019 and 2022. Non-COVID-19-related papers are measured against the left axis; COVID-19-related papers are measured against the right axis. Note: the left and right axes are on different (1:10) scales. (C) Kaplan–Meier plot showing time-to-event curves for peer-reviewed publication of preprints. Results are presented for COVID-19 preprints, non-COVID-19 preprints during the pandemic, and preprints before the pandemic.

Peer-reviewed COVID-19 articles were published at a steady rate (∼10 000/month) from May 2020 (Figure 2A). COVID-19 preprints spiked to a high of >4000/month in May 2020 but declined steadily over the remainder of the study period (Figure 2B). Non-COVID-19 journal article publication rates fell during the pandemic period compared with the pre-pandemic era, with the number of papers published per month increasing by 7260 each month in 2019 and by only 1385 papers each month thereafter (Supplementary Figure S1; rate change model *P* = 0.03). We did not identify a change in the slope between 2019 and 2020–2 in non-COVID-19 preprints (*P* = 0.23) (Supplementary Figure S.1B). Sensitivity analyses showed declines in non-COVID-19 publishing rates when the corpus was restricted to medical journal articles and bioRxiv/medRxiv preprints (Supplementary Figure S2).

### Attention analyses

#### Scientific citations

COVID-19 journal articles had received an average 16.0 (S.D. 131.76) citations and COVID-19 preprints 5.30 (S.D. 22.94) citations compared with 4.05 (S.D. 18.85) and 0.30 (S.D. 2.3) respectively for non-COVID-19 journal articles and for non-COVID-19 preprints from the same period, and 9.87 (S.D. 31.23) and 0.58 (S.D. 3.46) for journal articles and preprints from 2019. The distribution of citations is highly skewed: a majority of papers received few or no citations and a small minority received a very large number (Supplementary Figure S3). The skew is more pronounced in COVID-19 papers and preprints: 2.6% of COVID-19 papers had received ≥100 citations vs 0.2% of non-COVID-19 papers during the same period (Supplementary Figure S3 and Table 1); 63.9% of COVID-19 papers and 51.2% of COVID-19 preprints had received at least one citation compared with 53.4% of non-COVID-19 papers and 12.2% of non-COVID-19 preprints from the same period.

The most highly cited COVID-19 papers are those that cover the early research into hospitalized patients in Wuhan from the first 3 months of 2020 (Huang *et al.* had received 33 137 citations at the time of export [15]) (Supplementary Table S1). The journals with the most citations for COVID-19 articles were *The New England Journal of Medicine* (258 605 citations, average 312.70 per COVID-19 paper) and *The Lancet* (228 287 citations, average 241.80) (Supplementary Table S2).

#### Altmetrics

COVID-19 research generated nearly 10 times the media and social media attention per paper than non-COVID-19-related research, either from 2019 or from 2020–2: the average Altmetrics score for a COVID-19 journal article was 38.31 (SD 383.52) compared with 3.88 (SD 46.06) for a non-COVID-19 article from 2020–2 and 4.29 (SD 44.07) for papers from 2019 (Table 1). Preprints also generated more attention than journal articles for COVID-19 research [the average Altmetrics score was 45.74 (SD 365.19) for COVID-19 preprints compared with 38.31 (SD 383.52) for published articles] and non-COVID-19 publications, with an average Altmetrics score of 4.08 (SD 35.38) for non-COVID-19 preprints compared with 3.88 (SD 46.06) for non-COVID-19 journal articles in 2020–2. As with citations, the distribution of Altmetrics scores is skewed, with most papers and preprints receiving little or no media or social media attention, but with a longer ‘tail’ on COVID-19 papers and preprints (Supplementary Figure S4).

**Figure 3.**
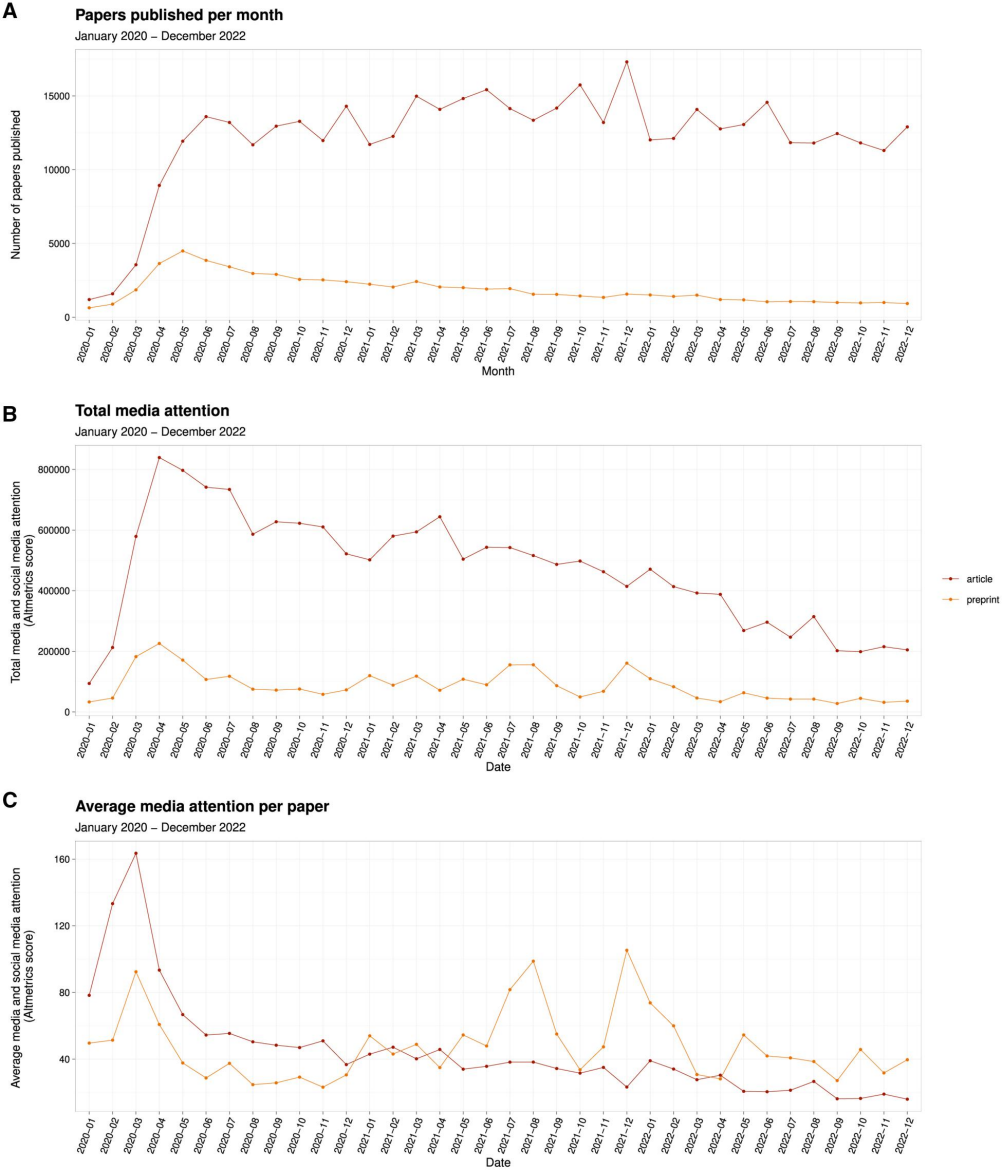
Publication rates, total media attention, and average media attention for COVID-19 papers, 2019–2. (A) Lines show the number of COVID-19 papers published per month for journal articles and preprints. (B) Lines show the monthly sum of media attention for journal articles and preprints. (C) Lines show the monthly average of media attention per paper (sum of media attention divided by number of papers) for journal articles and preprints.

Per-paper attention for journal articles peaked in the first wave (early 2020) and then declined to one-tenth of the first-wave level, with no peaks in between (Figure 3C). The per-paper attention for preprints saw two peaks of equal size to the first-wave peak, again corresponding with the Delta and Omicron variants.

### Correlations between scientific attention and media attention

**Figure 4.**
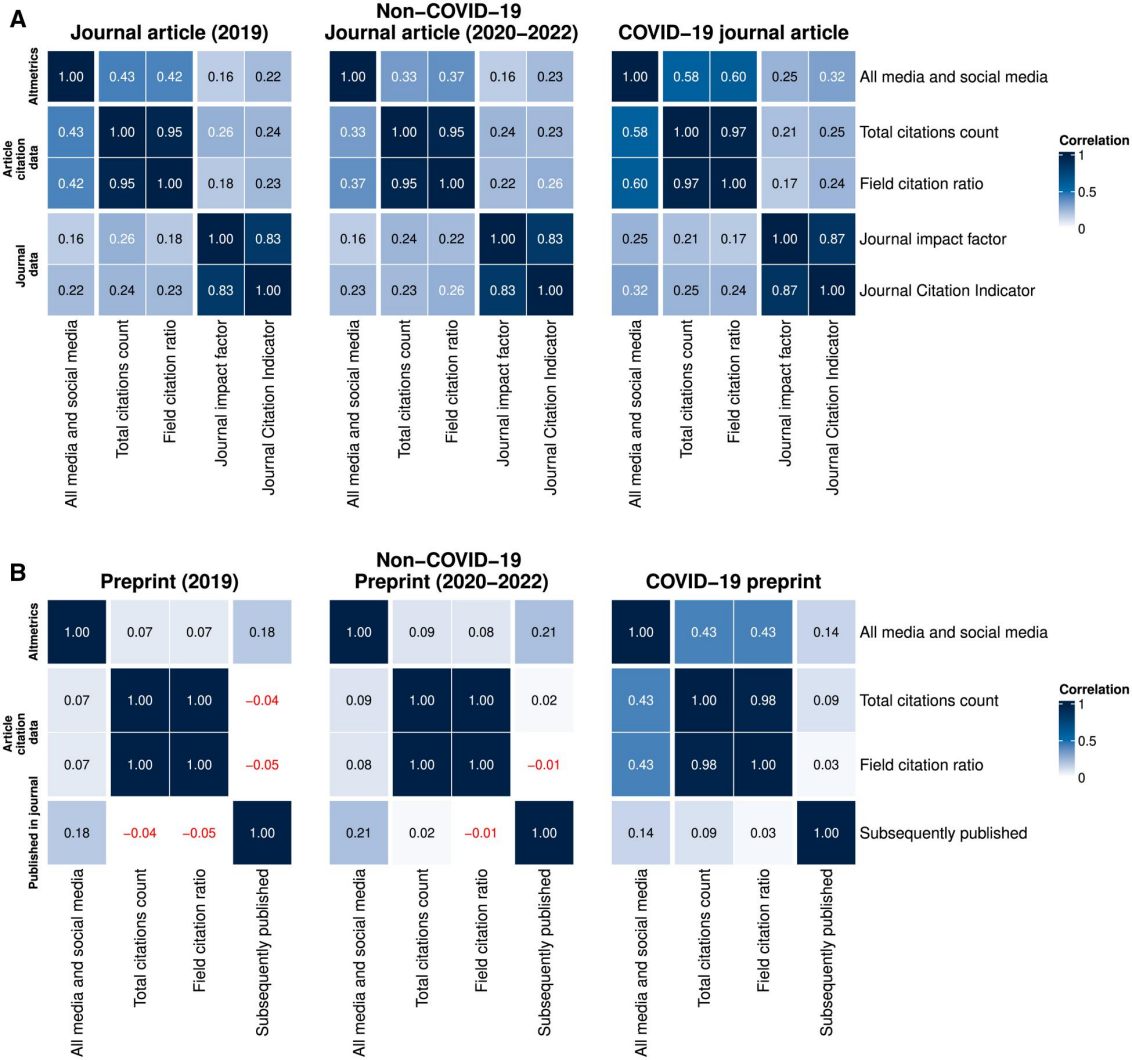
Heat maps showing the correlation between article citation metrics and journal impact metrics with media attention, as measured by using the article Altmetrics score. (A) Correlations for journal articles, stratified by pre-pandemic/non-COVID-19/COVID-19 status. (B) Correlations for preprints, stratified by pre-pandemic/non-COVID-19/COVID-19 status.

For COVID-19 journal articles, we observed a much stronger correlation between citation metrics and the Altmetrics score than for non-COVID-19 articles or pre-pandemic articles (Figure 4A). We observed a 0.58 correlation between the aggregated Altmetrics score and the citation count for COVID-19 journal articles—stronger than that for pre-pandemic journal articles (0.43) and non-COVID-19 journal articles from 2020–2 (0.33). The same holds true for the field citation ratio, and for the Journal Impact Factor and Journal Citation Indicator.

For preprints, there was a stronger correlation between most of the citation-based metrics of scientific attention and the Altmetrics score for COVID-19 preprints than for non-COVID-19 preprints or pre-pandemic preprints. We observed a 0.43 correlation between citation count and Altmetrics score for COVID-19 preprints: 0.07 for pre-pandemic journal articles and 0.09 for non-COVID-19 journal articles from 2020–2. However, the correlations between the Altmetrics score and the chance of subsequent publication were lower for COVID-19 preprints than for non-COVID-19 preprints from 2020–2 and pre-pandemic preprints.

### Subsequent publication of preprints

Time-to-event analysis modelling the ‘hazard’ of subsequent peer-reviewed publication showed that COVID-19 preprints have a lower probability of publication after preprinting than non-COVID-19 preprints, from either 2019 or 2020–2 (Figure 2C). One year after preprinting, 22.0% of COVID-19 preprints had been published vs 22.6% of non-COVID-19 preprints from 2020–2, and 32.2% of preprints from 2019 (Table 1). Cox proportional-hazards models estimated (i) a 62% greater ‘hazard’ of peer-reviewed publication for preprints prior to the pandemic (during 2019) compared with COVID-19 preprints, with a hazard ratio (HR) of 1.62 (95% confidence interval (CI): 1.60, 1.65), and (ii) a 19% higher hazard of publication for non-COVID-19 preprints during the pandemic compared with COVID-19 preprints, with an HR of 1.19 (95% CI: 1.17, 1.21). Restricting our analysis to bioRxiv and medRxiv preprints yielded similar conclusions (Supplementary Figure S5).

Further constraining our analysis to only preprints from 1 January to 1 August 2020 (*N* = 276 599), when the large spike of COVID-19 preprints appeared, showed a similar pattern with greater differences in subsequent publication rates (Supplementary Figure S6): non-COVID-19 preprints had a 76% higher hazard of publication (HR 1.76; 95% CI: 1.71,1.81) and non-COVID-19 medRxiv/bioRxiv preprints had a 90% higher hazard of publication (HR 1.90; 95% CI: 1.82,1.98).

### Topic modelling

**Figure 5.**
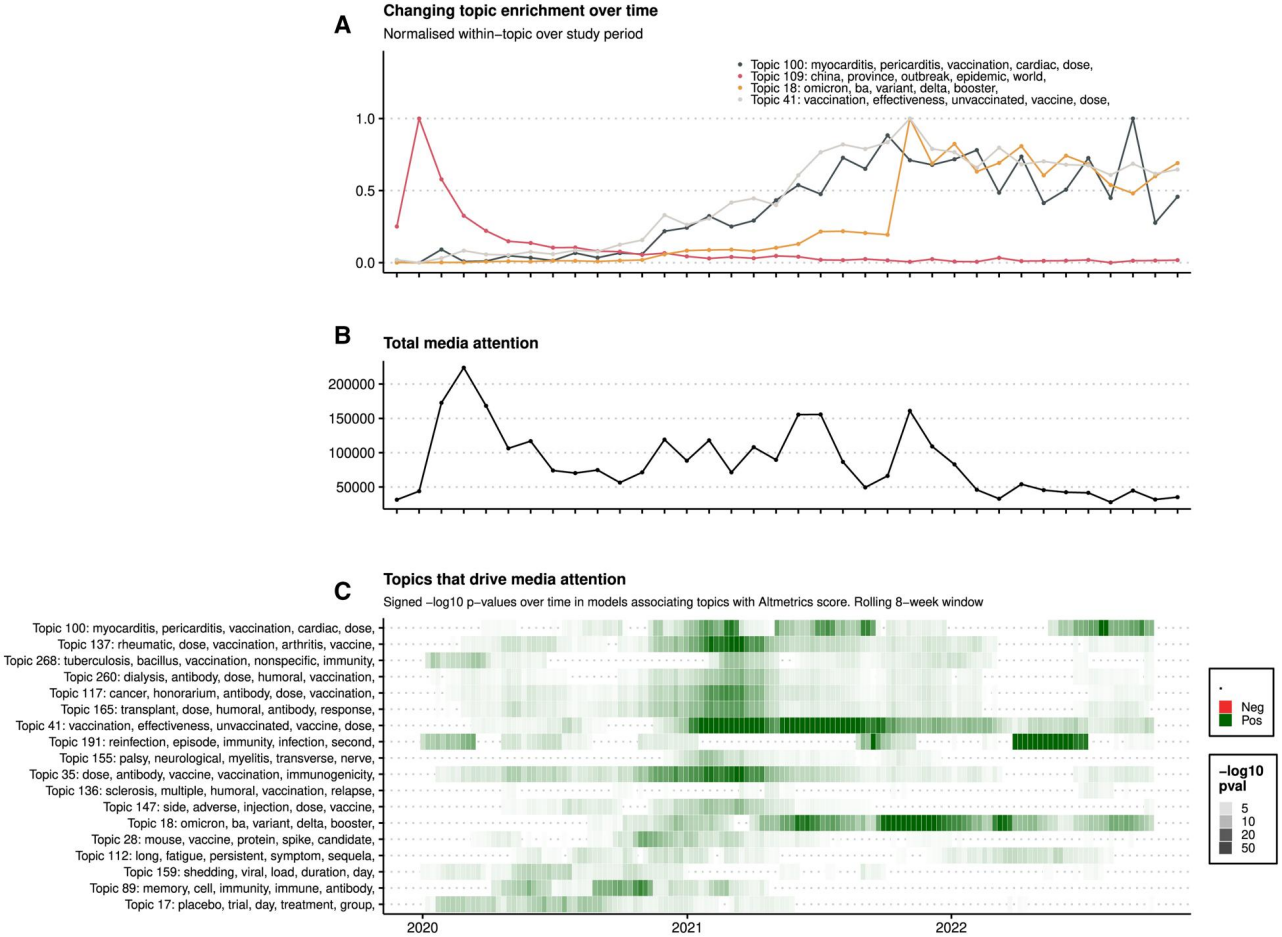
Preprints. (A) Line plot showing changes in the relative enrichment of preprint abstracts in four topics of particular public health interest over time. Enrichment is quantified as the mean probability value for each topic across the abstracts published in a given month. (B) Line plot showing total media attention paid to preprints across the study period, shown as a percentage of the peak level. (C) Heat map showing results of univariable regression of Altmetrics scores onto abstract topic probabilities on a rolling 8-week time window throughout the pandemic period 2020–2 among preprints only. Colour represents beta-values and colour darkness represents –log10 P-value (darker hue = lower *P*-value). Topics are ordered by average beta-value across the 3 years and only topics with at least one period of association (*P* < 0.05) with Altmetrics scores are shown.

A total of 299 latent topics were identified by using the BERTopic algorithm (Supplementary Table S3). Aggregated topic enrichment revealed evolving areas of research focus over time and more rapid changes in topic enrichment in preprints relative to journal articles. In journal articles, for instance, enrichment in Topic 18 (Omicron, ba, variant, delta, booster …) preprints rises steadily from the end of 2021 and the advent of the Omicron variant (Supplementary Figure S7); in preprints, by contrast, the topic enrichment spikes sharply at the end of 2021 and remains high for the rest of the pandemic period (Figure 5C).

In linear regression models, the strongest positive associations with the Altmetrics score were Topic 41 (vaccination, effectiveness, unvaccinated, vaccine, dose …), Topic 35 (dose, antibody, vaccine, vaccination, immunogenicity …) and Topic 100 (myocarditis, pericarditis, vaccination, cardiac, dose …) (Supplementary Figure S9). The strongest associations with citation rates were Topic 30 (China, December, respiratory, virus, syndrome …) and Topic 189 (host, epithelial, expression, innate, lung …) (Supplementary Figure S9). While there was generally a high degree of consistency between the drivers of citation and the drivers of attention [the effect direction of the associations was consistent in 250 of 299 (83.6%) of the *P* < 0.05 associations], some topics stood out as having different effects: Topics 235, 82, and 210 all related to mask wearing and showed positive associations (*P* < 0.05) with Altmetrics scores and negative associations with citation rates (Supplementary Figure S8C).

The time-varying association analysis among preprints (Figure 5C) revealed spikes of strong association between particular topics and media attention. For example, spikes in Topic 18 (which captures the Omicron and Delta variants) coincide with the emergence of the Delta variant (in mid-2021) and the Omicron variants (in late 2021 and early 2022) (Figure 5C). The same effect is less apparent in journal articles (Supplementary Figures S7 and S8), for which the strength of the topic–Altmetrics associations is less variable over the pandemic period.

## Discussion

Using preprints and peer-reviewed articles from the beginning of 2019 to the end of 2022, we describe a broad and dynamic response to COVID-19 from the research community, comprising >508 000 research papers and constituting 3.7% of the peer-reviewed research output in English between 2020 and 2022. Preprints played a significant role in the pandemic publishing ecosystem, with a spike in COVID-19 preprints in 2020 and increased use of preprints by researchers, the general public, and the news media. More generally, we describe a massively elevated level of public interest in published scientific research on COVID-19, and increased levels of citation, relative to non-COVID-19 research.

The increase in the release rate of preprints was most pronounced in a large ‘first wave’ of preprinted articles between January and August 2020. In time-to-event analysis, COVID-19 preprints were found to be less likely to get published than preprints on other subjects, from either before or during the pandemic, and this effect was more pronounced in preprints from this initial wave. This may indicate changing motivations for preprinting—e.g. using preprints to rapidly publish results with no intention of submitting them for subsequent publication. It may also indicate that the quality of COVID-19 preprints was, for a time, lower than the normal average quality for a preprint; or that the volume of COVID-19 preprints had hit a ‘ceiling’ of available publishing resources in journals; or that researchers had moved onto other topics during COVID-19 given the rapidly changing landscape. Our sensitivity analysis found that COVID-19 preprints on medRxiv and bioRxiv, especially those from the ‘first wave’, had a higher likelihood of publication in the first ∼200 days after preprinting, suggesting that early movers on the most visible preprint platforms had more chance of publication.

COVID-19 papers received an order of magnitude more citations and public/media attention than non-COVID-19 research. For both preprints and peer-reviewed papers, the maximum point of per-paper public interest (Altmetrics) was in March 2020, at the height of the ’first wave’ of the pandemic; however, while per-paper interest steadily declined for peer-reviewed papers thereafter, preprints experienced second and third ‘waves’ of interest that corresponded with the emergence of new SARS-CoV-2 variants in summer 2021 (Delta) and winter 2021–2 (Omicron). These spikes of interest were associated with elevated interest in specific research topics that were relevant to contemporaneous areas of public health interest, as demonstrated by the waxing and waning strengths of association in the time-varying topic modelling association analysis, and the spikes are more apparent in preprints than in peer-reviewed papers. This alignment is likely to be bidirectional: research both drives the public discussion and is driven by it, and these results cannot disambiguate the direction of effect. Whichever the effect direction, preprints are more responsive to and instigative of public interest because they are disseminated faster. This effect is naturally heightened during a pandemic when novel information is at a premium, and concern that non-peer-reviewed work may be driving public opinion and discussion has led to calls for a more robust set of guidelines for the discussion of preprint research by the media [5, 16–18]. This need will only become more acute, given the moves by funding bodies [19] and journals [20] to increase the role of preprints in scientific publishing.

We found that the correlation between the metrics of scientific attention and Altmetrics was higher in COVID-19 articles and preprints than in non-COVID-19 ones. This suggests that, for COVID-19 articles, there was greater alignment between scientific interest and public attention. It may be that papers with intrinsic scientific merit gained more attention, were more highly cited, and were more likely to be published in higher-impact-factor journals. Conversely, media and social media attention could have increased a paper’s chances of being cited or published in higher-impact journals, especially for COVID-19 papers. Previous research has framed Altmetrics impact as being ‘predictive’ of higher citations [21] but causality has not been investigated and cannot be inferred from this analysis.

Previous research has described the rapid redirection of research efforts into COVID-19 [8] and suggested that the pandemic may have adversely impacted scientific output in non-COVID-19 fields [9, 10]. We find indicative evidence of a decline in the growth rate of non-COVID-19-related scientific output throughout the pandemic period.

## Limitations

Linkage of preprints to subsequent peer-reviewed publications may be incomplete, resulting in biased estimates of publication rates and predictors of publication. Granular Altmetrics data were not available for all papers owing to computational limitations. The Dimensions dataset, while extensive, does not capture 100% of the research literature. Our analysis does not account for variations in preprinted vs published versions of articles, which may vary between paper types and influence subsequent publication rates. Our analysis ends in 2022, before the ‘recovery’ phase of the pandemic. The permanence or otherwise of the described disruptions is therefore a matter for future research.

## Conclusion

In conclusion, we describe a large response from the scientific community to the COVID-19 pandemic and a substantial increase in citations and public attention, especially for preprints. Evolving research topics were aligned with evolving areas of public interest and may partly be driven by them. These findings contribute to an understanding of how scientific discourse functions during a pandemic—particularly on the relationship between scientific discourse and the media, and on the role of preprints in the research ecosystem.

## Ethics approval

This study did not require any ethical approval.

## Supplementary Material

dyaf058_Supplementary_Data

## Data Availability

The data used in this study were provided by Dimensions under proposal DIM-251. Applications for access to the data should be made to Dimensions. Code can be accessed here: https://github.com/mathzero/covid_lit_analysis.
