# Supplementary material for: How COVID-19 affected academic publishing: a 3-year study of 17 million research papers
Source: Int J Epidemiol. 2025 May 27;54(3):dyaf058. doi: 10.1093/ije/dyaf058 (PMC12107239; doi:10.1093/ije/dyaf058)
Supplement: dyaf058_Supplementary_Data [file dyaf058_supplementary_data.pdf]

How COVID-19 affected academic publishing: a three-year study of 17 million research papers

Matthew Whitaker PhD, Sabrina Rodrigues PhD, Graham Cooke PhD, Bérangère Virlon PhD, Christl A Donnelly ScD, Helen Ward PhD, Paul Elliott PhD, and Marc Chadeau-Hyam PhD

Supplementary Material

Table S.1: Top 20 COVID-19 papers, ranked by citation count (A) and Altmetrics score (B). Two papers which appear in both tables are highlighted in bold.

A

Title	Journal	Publication date	Altmetrics score	Citations
Clinical features of patients infected with 2019 novel coronavirus in Wuhan, China	*The Lancet*	01/24/2020	14351	35137
Clinical Characteristics of Coronavirus Disease 2019 in China	*New England Journal of Medicine*	02/28/2020	9994	21948
A Novel Coronavirus from Patients with Pneumonia in China, 2019	*New England Journal of Medicine*	01/24/2020	8111	20663
Clinical course and risk factors for mortality of adult inpatients with COVID-19 in Wuhan, China: a retrospective cohort study	*The Lancet*	03/11/2020	12930	20200
Clinical Characteristics of 138 Hospitalized Patients With 2019 Novel Coronavirus–Infected Pneumonia in Wuhan, China	*JAMA*	03/17/2020	6354	17774
A pneumonia outbreak associated with a new coronavirus of probable bat origin	*Nature*	02/03/2020	7431	15737
Epidemiological and clinical characteristics of 99 cases of 2019 novel coronavirus pneumonia in Wuhan, China: a descriptive study	*The Lancet*	01/30/2020	4310	15367
SARS-CoV-2 Cell Entry Depends on ACE2 and TMPRSS2 and Is Blocked by a Clinically Proven Protease Inhibitor	*Cell*	03/05/2020	4171	14437
Characteristics of and Important Lessons From the Coronavirus Disease 2019 (COVID-19) Outbreak in China	*JAMA*	04/07/2020	10869	13743
Early Transmission Dynamics in Wuhan, China, of Novel Coronavirus–Infected Pneumonia	*New England Journal of Medicine*	01/29/2020	6753	12263
**Safety and Efficacy of the BNT162b2 mRNA Covid-19 Vaccine**	***New England Journal of Medicine***	**12/10/2020**	**30736**	**9626**
Genomic characterisation and epidemiology of 2019 novel coronavirus: implications for virus origins and receptor binding	*The Lancet*	01/30/2020	4191	8774
A new coronavirus associated with human respiratory disease in China	*Nature*	02/03/2020	2974	8449
Dexamethasone in Hospitalized Patients with Covid-19	*New England Journal of Medicine*	07/17/2020	9337	7843
An interactive web-based dashboard to track COVID-19 in real time	*The Lancet Infectious Diseases*	02/19/2020	932	7773

Clinical course and outcomes of critically ill patients with SARS-CoV-2 pneumonia in Wuhan, China: a single-centered, retrospective, observational study	*The Lancet Respiratory Medicine*	02/24/2020	3435	7619
COVID-19: consider cytokine storm syndromes and immunosuppression	*The Lancet*	03/16/2020	2809	7091
Presenting Characteristics, Comorbidities, and Outcomes Among 5700 Patients Hospitalized With COVID-19 in the New York City Area	*JAMA*	05/26/2020	7649	7074
Cryo-EM structure of the 2019-nCoV spike in the prefusion conformation	*Science*	02/19/2020	4084	6937
**Aerosol and Surface Stability of SARS-CoV-2 as Compared with SARS-CoV-1**	***New England Journal of Medicine***	**03/17/2020**	**24478**	**6858**

B

Title	Journal	Publication date	Altmetrics score	Citations
Covid-19: Researcher blows the whistle on data integrity issues in Pfizer's vaccine trial	*The BMJ*	11/02/2021	45148	30
The proximal origin of SARS-CoV-2	*Nature Medicine*	03/17/2020	35361	3603
Effectiveness of Adding a Mask Recommendation to Other Public Health Measures to Prevent SARS-CoV-2 Infection in Danish Mask Wearers	*Annals of Internal Medicine*	11/18/2020	33429	243
Comparing SARS-CoV-2 natural immunity to vaccine-induced immunity: reinfections versus breakthrough infections	*medRxiv*	08/25/2021	32810	146
Ivermectin for Prevention and Treatment of COVID-19 Infection: A Systematic Review, Meta-analysis, and Trial Sequential Analysis to Inform Clinical Guidelines	*American Journal of Therapeutics*	06/21/2021	31303	158
**Safety and Efficacy of the BNT162b2 mRNA Covid-19 Vaccine**	***New England Journal of Medicine***	**12/10/2020**	**30736**	**9626**
Intracellular Reverse Transcription of Pfizer BioNTech COVID-19 mRNA Vaccine BNT162b2 In Vitro in Human Liver Cell Line	*Current Issues in Molecular Biology*	02/25/2022	27841	33
Safety and efficacy of an rAd26 and rAd5 vector-based heterologous prime-boost COVID-19 vaccine: an interim analysis of a randomised controlled phase 3 trial in Russia	*The Lancet*	02/02/2021	27159	1212
COVID UPDATE: What is the truth?	*Surgical Neurology International*	04/22/2022	26679	5
COVID-19: stigmatising the unvaccinated is not justified	*The Lancet*	11/18/2021	24860	40
**Aerosol and Surface Stability of SARS-CoV-2 as Compared with SARS-CoV-1**	***New England Journal of Medicine***	**03/17/2020**	**24478**	**6858**
Increases in COVID-19 are unrelated to levels of vaccination across 68 countries and 2947 counties in the United States	*European Journal of Epidemiology*	09/30/2021	24072	59
Physical distancing, face masks, and eye protection to prevent person-to-person transmission of SARS-CoV-2 and COVID-19: a systematic review and meta-analysis	*The Lancet*	06/01/2020	24020	2801
The Incidence of Myocarditis and Pericarditis in Post COVID-19 Unvaccinated Patients—A Large Population-Based Study	*Journal of Clinical Medicine*	04/15/2022	22940	12
The epidemiological relevance of the COVID-19-vaccinated population is increasing	*The Lancet Regional Health - Europe*	11/20/2021	22575	18

The FDA-approved drug ivermectin inhibits the replication of SARS-CoV-2 in vitro	*Antiviral Research*	04/03/2020	22108	1457
RETRACTED: Hydroxychloroquine or chloroquine with or without a macrolide for treatment of COVID-19: a multinational registry analysis	*The Lancet*	05/22/2020	22051	806
Post-lockdown SARS-CoV-2 nucleic acid screening in nearly ten million residents of Wuhan, China	*Nature Communications*	11/20/2020	22025	138
Ten scientific reasons in support of airborne transmission of SARS-CoV-2	*The Lancet*	04/15/2021	21623	538
An evidence review of face masks against COVID-19	*Proceedings of the National Academy of Sciences of the United States of America*	01/11/2021	20797	710

Table S.2: Top 10 journals, ranked by citation count to COVID-19 papers only.

Journal	N papers published	Mean Altmetric score	Total citations	Mean per-paper citations	Journal Impact Factor
New England Journal of Medicine	827	1235.80	258605	312.70	176.08
The Lancet	944	937.70	228287	241.80	202.73
JAMA	890	692.50	152324	171.20	157.34
Nature	1205	882.80	146442	121.50	69.50
Science	656	788.40	110444	168.40	63.71
Cell	196	813.60	89420	456.20	66.85
International Journal of Environmental Research and Public Health	5321	9.30	85727	16.10	-
PLOS ONE	4267	36.90	83064	19.50	2.90
The BMJ	2752	274.70	77279	28.10	93.6
Clinical Infectious Diseases	1250	176.20	75613	60.50	21.00

Table S.3: 299 topics identified by Bertopic topic modelling of abstracts from 508,436 COVID-19 papers published in 2020-2022. The top 10 terms associated with the topic (as measured by probability value) are shown for each topic.

Topic	Words
0	learning, teaching, education, school, student, educational, distance, class, virtual, academic
1	detection, assay, sensitivity, testing, diagnostic, specificity, rapid, antigen, test, amplification
2	vaccine, hesitancy, vaccination, acceptance, uptake, willingness, intention, vaccinate, get, receive
3	political, legal, governance, law, government, policy, public, international, article, crisis
4	anxiety, mental, depression, psychological, stress, health, depressive, distress, fear, disorder
5	model, forecasting, epidemic, forecast, mathematical, prediction, number, reproduction, equilibrium, sir
6	thrombosis, thrombotic, venous, coagulation, embolism, bleeding, heparin, pulmonary, platelet, anticoagulant
7	protease, pro, binding, molecular, drug, inhibitor, energy, antiviral, affinity, compound
8	dental, oral, dentistry, orthodontic, periodontal, practice, knowledge, cavity, mouth, periodontitis
9	genome, genomic, mutation, phylogenetic, lineage, sequence, variant, evolution, genetic, evolutionary
10	food, insecurity, agricultural, security, supply, agriculture, production, meat, nutrition, consumption
11	work, job, working, employee, leadership, organizational, gender, home, productivity, satisfaction
12	chest, lung, pneumonia, consolidation, tomography, score, pulmonary, pleural, involvement, scan
13	neurological, brain, nervous, encephalopathy, encephalitis, central, cerebrospinal, acute, syndrome, system
14	spike, binding, ace, protein, receptor, fusion, entry, domain, affinity, host
15	deep, accuracy, chest, classification, neural, image, convolutional, network, learning, detection
16	sleep, insomnia, quality, anxiety, depression, stress, mental, psychological, poor, disturbance
17	placebo, trial, day, treatment, group, arm, standard, efficacy, therapy, adverse
18	omicron, ba, variant, delta, booster, spike, neutralization, escape, vaccination, dose
19	diabetes, glucose, diabetic, insulin, hyperglycemia, td, type, nondiabetic, mortality, blood
20	hypertension, mortality, diabetes, death, age, chronic, risk, admission, hypertensive, disease
21	air, pollution, quality, particulate, emission, ozone, concentration, pollutant, atmospheric, traffic
22	immune, storm, inflammatory, innate, activation, cell, inflammation, expression, severe, response
23	cardiac, myocardial, injury, myocarditis, cardiovascular, ventricular, heart, left, coronary, dysfunction

Topic	Words
24	knowledge, attitude, practice, good, towards, awareness, preventive, prevention, questionnaire, level
25	care, virtual, satisfaction, video, telephone, patient, remote, consultation, visit, primary
26	tourism, tourist, travel, industry, destination, sector, hospitality, domestic, crisis, recovery
27	older, elderly, aging, social, isolation, people, life, living, aged, mental
28	mouse, vaccine, protein, spike, candidate, intranasal, immunogenicity, immune, mucosal, induced
29	youth, child, parental, young, mental, family, adolescent, stress, parent, emotional
30	china, december, respiratory, virus, syndrome, world, corona, novel, pneumonia, spread
31	pregnant, pregnancy, maternal, neonatal, birth, delivery, fetal, gestational, trimester, obstetric
32	physical, pa, activity, exercise, sedentary, fitness, active, sport, inactivity, sitting
33	surgical, surgery, elective, postoperative, preoperative, emergency, operating, underwent, plastic, hospital
34	rehabilitation, exercise, physiotherapy, muscle, functional, physical, discharge, strength, pulmonary, function
35	dose, antibody, vaccine, vaccination, immunogenicity, booster, humoral, response, second, immune
36	ventilation, prone, intubation, oxygen, mechanical, failure, oxygenation, respiratory, invasive, rox
37	stock, market, volatility, index, financial, trading, price, return, exchange, investor
38	olfactory, smell, taste, anosmia, dysfunction, od, gustatory, loss, hyposmia, recovery
39	liver, injury, cirrhosis, ast, alt, hepatitis, hepatic, bilirubin, abnormal, alanine
40	transplant, transplantation, kidney, sot, organ, donor, lung, graft, solid, recipient
41	vaccination, effectiveness, unvaccinated, vaccine, dose, breakthrough, booster, fully, hospitalization, aged
42	burnout, exhaustion, emotional, depersonalization, inventory, stress, job, accomplishment, personal, working
43	kidney, renal, injury, creatinine, proteinuria, tubular, acute, replacement, glomerular, mortality
44	transport, travel, transportation, transit, traffic, mobility, bus, cycling, road, passenger
45	antibiotic, bacterial, resistance, bacteria, stewardship, aureus, klebsiella, staphylococcus, resistant, pseudomonas
46	mucormycosis, fungal, cam, orbital, diabetes, fungus, sinus, invasive, mucorales, opportunistic
47	rheumatic, arthritis, ra, rheumatoid, none, lupus, declared, systemic, antirheumatic, inflammatory
48	vitamin, deficiency, supplementation, serum, deficient, severity, oh, mortality, hypovitaminosis, status
49	pediatric, fever, cough, clinical, respiratory, asymptomatic, mild, laboratory, chest, severe
50	pregnant, anxiety, pregnancy, depression, maternal, prenatal, postnatal, stress, mental, birth
51	air, indoor, aerosol, ventilation, transmission, room, contamination, environmental, droplet, particle
52	bat, dog, specie, animal, zoonotic, mink, deer, wildlife, wild, human
53	marketing, consumer, shopping, customer, brand, purchase, behavior, advertising, intention, behaviour
54	occupational, exposure, workplace, staff, risk, working, personnel, contact, work,

Topic	Words
	infection
55	loneliness, social, lonely, isolation, older, mental, depression, depressive, living, anxiety
56	obesity, obese, adipose, overweight, metabolic, weight, fat, body, tissue, mass
57	financial, banking, credit, bank, profitability, liquidity, capital, corporate, islamic, loan
58	nursing, nurse, care, staff, leadership, professional, qualitative, practice, working, work
59	coping, resilience, stress, psychological, anxiety, mental, scale, depression, emotional, support
60	temperature, humidity, meteorological, weather, wind, climate, daily, relative, climatic, correlation
61	sport, football, training, season, soccer, sporting, professional, athletic, league, elite
62	excess, mortality, death, expectancy, fatality, life, age, estimate, lost, population
63	genetic, allele, gene, locus, ace, susceptibility, polymorphism, genotype, epigenetic, host
64	saliva, nasopharyngeal, salivary, detection, specimen, swab, testing, sensitivity, collection, diagnostic
65	coronary, infarction, myocardial, ami, cardiac, cardiovascular, heart, cardiology, percutaneous, period
66	cancer, anticancer, chemotherapy, lung, mortality, malignancy, solid, death, treatment, risk
67	score, validation, prediction, nomogram, curve, calibration, mortality, admission, predictive, cohort
68	energy, electricity, renewable, consumption, carbon, demand, green, power, economic, sector
69	sentiment, twitter, medium, topic, opinion, tweet, public, neutral, machine, social
70	supply, chain, logistics, resilience, demand, supplier, industry, disruption, production, inventory
71	privacy, tracing, contact, digital, security, mobile, adoption, technology, user, surveillance
72	sexual, sex, syphilis, men, prep, sexually, erectile, gay, bisexual, sexuality
73	maternity, birth, pregnant, maternal, antenatal, pregnancy, prenatal, care, obstetric, neonatal
74	unemployment, employment, labour, labor, market, job, wage, earnings, unemployed, economic
75	religious, church, religion, worship, islamic, faith, theological, god, christian, islam
76	plasma, convalescent, transfusion, therapy, titer, antibody, treatment, day, donor, efficacy
77	cancer, oncology, care, virtual, patient, treatment, chemotherapy, delivery, access, pandemic
78	waste, plastic, disposal, management, solid, environmental, generation, pollution, face, pyrolysis
79	black, white, hispanic, ethnic, racial, race, asian, minority, mortality, american
80	violence, domestic, intimate, abuse, partner, sexual, assault, police, violent, victimization
81	thrombocytopenia, thrombosis, platelet, thrombotic, venous, cerebral, vaccination, vaccine, rare, sinus
82	mask, face, wearing, wear, use, public, usage, cloth, facial, wore
83	eye, ophthalmology, ophthalmic, cataract, visual, glaucoma, ocular, retinal, macular, acuity
84	disinfection, inactivation, decontamination, irradiation, ozone, ultraviolet, surface, inactivate, virucidal, log

Topic	Words
85	suicide, suicidal, ideation, mental, psychiatric, si, attempt, depression, increase, risk
86	dementia, cognitive, living, people, care, older, family, impairment, support, neuropsychiatric
87	urban, green, space, outdoor, park, recreation, city, nature, housing, street
88	tracheostomy, intubation, tracheal, airway, tracheotomy, percutaneous, procedure, ventilation, mechanical, decannulation
89	memory, cell, immunity, immune, antibody, humoral, response, convalescent, effector, spike
90	scientific, research, publication, journal, science, citation, collaboration, literature, top, scopus
91	spatial, spatiotemporal, density, migration, epidemic, spread, population, diffusion, urban, local
92	pregnant, pregnancy, maternal, vaccination, vaccine, nonpregnant, acceptance, unvaccinated, birth, neonatal
93	business, small, strategic, micro, economic, company, research, crisis, continuity, competitiveness
94	asthma, allergic, asthmatic, rhinitis, allergy, ait, exacerbation, atopic, ar, pollen
95	machine, prediction, accuracy, predict, model, learning, classifier, forest, predictive, algorithm
96	chloroquine, treatment, drug, antimalarial, antiviral, efficacy, prolongation, clinical, safety, adverse
97	pain, chronic, back, neck, neuropathic, intensity, physical, disability, knee, va
98	influenza, seasonal, season, respiratory, syncytial, winter, flu, circulation, virus, surveillance
99	cognitive, neuropsychiatric, impairment, brain, memory, neuropsychological, neurological, executive, fog, cognition
100	myocarditis, pericarditis, vaccination, cardiac, dose, vaccine, adverse, rare, following, second
101	skin, cutaneous, rash, maculopapular, urticaria, dermatological, urticarial, erythema, vesicular, livedo
102	aspergillosis, fungal, aspergillus, invasive, pulmonary, bacterial, ill, critically, lavage, specie
103	trauma, orthopedic, injury, fracture, surgery, period, ankle, hand, decrease, emergency
104	tuberculosis, mycobacterium, pulmonary, treatment, diagnosis, incidence, notification, case, latent, sputum
105	urology, urological, prostate, bladder, urinary, urologic, stone, cancer, surgical, surgery
106	sewage, water, sludge, surveillance, concentration, feces, sewer, influent, detection, viral
107	vaccination, vaccine, coverage, allocation, model, immunity, strategy, optimal, would, population
108	hearing, tinnitus, vestibular, loss, vertigo, auditory, voice, speech, ear, vocal
109	china, province, outbreak, epidemic, world, spread, january, december, city, control
110	gut, microbiota, intestinal, gastrointestinal, microbial, fecal, composition, immune, tract, bacteria
111	mobility, spatial, human, travel, mobile, movement, data, retail, transit, residential
112	long, fatigue, persistent, symptom, sequela, dyspnea, pain, prevalence, illness, headache
113	psychiatric, schizophrenia, psychotic, psychosis, disorder, psychiatry, mental, bipolar, inpatient, neuropsychiatric

Topic	Words
114	college, stress, academic, depression, university, mental, anxiety, psychological, undergraduate, student
115	migration, migrant, asylum, refugee, immigration, labor, labour, international, border, repatriation
116	inflammatory, syndrome, shock, pediatric, fever, intravenous, rash, gastrointestinal, temporally, coronary
117	cancer, honorarium, antibody, dose, vaccination, response, chemotherapy, vaccine, solid, lymphoma
118	economic, economy, sri, growth, trade, world, global, impact, recession, sector
119	herbal, medicinal, antiviral, plant, extract, natural, medicine, vol, leaf, activity
120	thyroid, thyroiditis, sat, subacute, hypothyroidism, thyrotoxicosis, hormone, hyperthyroidism, gland, graf
121	alcohol, drinking, consumption, use, binge, alcoholic, heavy, risky, frequency, drink
122	count, lymphocyte, hematological, platelet, ratio, severity, blood, laboratory, lymphopenia, prognostic
123	abortion, fertility, contraceptive, contraception, reproductive, infertility, infertile, sexual, access, pregnancy
124	cough, fever, clinical, common, laboratory, throat, sore, epidemiological, mild, symptom
125	pneumothorax, spontaneous, emphysema, subcutaneous, complication, chest, tube, ventilation, pneumonia, pleural
126	brazil, brazilian, de, rio, mortality, state, northeast, lethality, socioeconomic, spatial
127	language, discourse, linguistic, speech, lexical, metaphor, english, corpus, vocabulary, semantic
128	weight, obesity, eating, gain, overweight, body, physical, activity, dietary, consumption
129	gi, gastrointestinal, diarrhea, digestive, abdominal, vomiting, nausea, tract, pain, intestinal
130	fibrosis, alveolar, pulmonary, lung, dad, autopsy, diffuse, damage, interstitial, fibrotic
131	ace, expression, ra, receptor, enzyme, entry, lung, adam, converting, cell
132	oxidative, mitochondrial, antioxidant, mitochondrion, redox, glutathione, stress, thiol, reactive, inflammation
133	breast, cancer, screening, surgery, stage, diagnosis, chemotherapy, cervical, treatment, endocrine
134	ocular, conjunctival, conjunctivitis, eye, conjunctiva, tear, surface, ophthalmic, swab, ophthalmological
135	stroke, ischemic, min, period, acute, time, emergency, care, arrival, hospital
136	sclerosis, multiple, humoral, vaccination, relapse, response, immune, therapy, dose, disability
137	rheumatic, dose, vaccination, arthritis, vaccine, ra, antibody, flare, none, bowel
138	cultural, solidarity, social, culture, collective, crisis, society, political, article, individualism
139	misinformation, fake, news, medium, false, information, content, social, twitter, conspiracy
140	frailty, frail, older, mortality, geriatric, age, elderly, score, admission, admitted
141	overdose, substance, drug, sud, harm, addiction, use, illicit, moud, treatment
142	stigma, stigmatization, discrimination, social, fear, people, scale, experienced, psychological, towards
143	ai, intelligence, artificial, technology, machine, big, fight, prediction, intelligent, paper
144	eating, ed, disorder, binge, weight, emotional, anorexia, food, disordered, stress
145	ed, emergency, department, ambulance, period, acuity, hospital, attendance, triage, reduction

Topic	Words
146	ethical, ethic, allocation, triage, ethically, moral, scarce, justice, research, guidance
147	side, adverse, injection, dose, vaccine, pain, headache, effect, site, vaccination
148	testosterone, sex, men, estrogen, hormone, male, androgen, estradiol, progesterone, female
149	dialysis, peritoneal, kidney, nephrology, renal, maintenance, incenter, chronic, care, unit
150	palliative, care, hospice, dying, symptom, team, consultation, specialist, inpatient, advance
151	de, da, em, para, brazil, pandemia, brazilian, do, na, um
152	pharmacy, pharmacist, pharmaceutical, medication, community, drug, service, practice, care, prescription
153	hotel, hospitality, industry, tourism, business, crisis, accommodation, customer, sector, marketing
154	nutritional, malnutrition, nutrition, enteral, intake, critically, ill, status, parenteral, stay
155	palsy, neurological, myelitis, transverse, nerve, vaccination, weakness, rare, vaccine, facial
156	semen, sperm, reproductive, testicular, testis, fertility, male, motility, men, ace
157	hand, hygiene, washing, soap, compliance, adherence, knowledge, practice, water, glove
158	extracorporeal, oxygenation, membrane, survival, refractory, failure, ventilation, support, day, initiation
159	shedding, viral, load, duration, day, onset, symptom, asymptomatic, clearance, positive
160	cruise, ship, maritime, port, shipping, crew, container, princess, diamond, industry
161	replication, translation, host, viral, protein, genome, complex, nonstructural, structure, phosphorylation
162	adherence, preventive, nonadherence, behavior, social, compliance, adhere, perception, physical, behaviour
163	appendicitis, aa, appendectomy, appendicectomy, complicated, perforated, surgical, surgery, acute, conservative
164	endoscopy, endoscopic, gastroenterology, gastrointestinal, gi, procedure, colonoscopy, equipment, endoscope, digestive
165	transplant, dose, humoral, antibody, response, kidney, vaccination, vaccine, third, solid
166	music, musical, dance, listening, singing, live, choir, mood, virtual, education
167	autism, autistic, spectrum, disorder, behavioral, child, parental, mental, developmental, school
168	discrimination, asian, racism, racial, hate, chinese, prejudice, american, xenophobia, racist
169	otolaryngology, surgery, ear, surgical, neck, otorhinolaryngology, tonsillitis, skull, rhinology, head
170	segmentation, dice, lesion, deep, lung, image, network, module, segment, tomography
171	stem, mesenchymal, therapy, cell, regenerative, therapeutic, tissue, treatment, regeneration, umbilical
172	zinc, selenium, se, supplementation, vitamin, deficiency, serum, copper, trace, immune
173	entrepreneurial, entrepreneurship, business, orientation, entrepreneur, innovation, crisis, intention, research, performance
174	fractional, numerical, derivative, model, uniqueness, mathematical, stability, solution, existence, operator
175	cancer, anxiety, distress, breast, depression, psychological, oncology, psychosocial, emotional, fear
176	validity, scale, psychometric, reliability, fear, factor, confirmatory, consistency, version,

Topic	Words
	item
177	smoking, tobacco, quit, cessation, cigarette, nicotine, smoke, use, hookah, dependence
178	grief, bereavement, mourning, funeral, grieving, loss, death, family, complicated, burial
179	bowel, colitis, inflammatory, ulcerative, biologic, disease, flare, hospitalization, therapy, remission
180	dengue, mosquito, fever, aedes, endemic, tropical, vector, febrile, diagnosis, serological
181	aviation, air, airport, passenger, flight, industry, transport, travel, aircraft, traffic
182	epilepsy, seizure, epileptic, frequency, antiepileptic, worsening, epileptiform, outpatient, people, depression
183	homelessness, homeless, shelter, housing, people, service, street, vulnerable, accommodation, substance
184	aging, senescence, elderly, expression, senescent, immune, aged, older, cellular, cell
185	indigenous, racism, black, racial, tribal, aboriginal, native, ethnic, american, structural
186	hip, fracture, femur, mortality, surgery, postoperative, proximal, femoral, day, stay
187	fishing, fish, aquaculture, shrimp, fishery, coastal, marine, production, sector, supply
188	quarantine, psychological, anxiety, depression, mental, stress, distress, depressive, emotional, health
189	host, epithelial, expression, innate, lung, cell, viral, gene, human, immune
190	diet, nutritional, dietary, immune, nutrition, food, intake, immunity, vitamin, system
191	reinfection, episode, immunity, infection, second, reactivation, positive, first, case, day
192	abo, blood, group, ab, type, susceptibility, association, severity, infection, risk
193	placental, placenta, fetal, maternal, pregnancy, villous, trophoblast, pregnant, trimester, expression
194	prison, correctional, detention, jail, incarceration, staff, probation, penitentiary, carceral, prisoner
195	trust, government, compliance, political, distrust, public, institutional, interpersonal, trustworthiness, crisis
196	malaria, falciparum, plasmodium, toxoplasmosis, endemic, elimination, burden, prevalence, antimalarial, incidence
197	corpus, text, retrieval, semantic, search, cord, literature, mining, scientific, language
198	neurosurgical, neurosurgery, elective, surgical, operative, spine, outpatient, training, practice, volume
199	endothelial, vascular, dysfunction, endothelium, activation, adhesion, inflammation, injury, plasma, damage
200	water, river, consumption, sanitation, quality, pollution, demand, drinking, heavy, supply
201	trade, export, foreign, international, trading, import, economic, supply, policy, china
202	library, academic, digital, university, copyright, information, access, reference, service, librarian
203	construction, industry, project, safety, sector, cost, site, productivity, management, impact
204	japan, japanese, prefecture, number, government, declaration, policy, emergency, april, state
205	antiviral, replication, drug, viral, activity, therapeutic, human, inhibit, epithelial, cell
206	moral, distress, morally, injury, utilitarian, ethical, mi, injurious, morality, personal
207	asymptomatic, prevalence, proportion, testing, estimate, population, symptomatic, infection, seropositive, positive
208	headache, migraine, frequency, intensity, symptom, pain, worsening, sleep, novo, aura
209	pediatric, ed, ped, emergency, department, utilization, period, decrease, visit, proportion
210	mask, face, wearing, detection, masked, deep, recognition, person, object, distance

Topic	Words
211	addiction, gaming, problematic, game, medium, use, social, excessive, disorder, usage
212	nosocomial, transmission, staff, hospital, ward, outbreak, infection, screening, cluster, control
213	uveitis, ocular, optic, retinal, eye, visual, vision, vaccination, anterior, acuity
214	complement, activation, ca, pathway, endothelial, coagulation, deposition, inflammatory, immune, thrombotic
215	lymphadenopathy, axillary, lymph, ipsilateral, node, breast, vaccination, adenopathy, cortical, supraclavicular
216	ethical, vaccination, legal, mandatory, vaccine, ethically, compulsory, court, law, right
217	cannabis, substance, alcohol, use, marijuana, tobacco, consumption, drinking, drug, cigarette
218	arrest, resuscitation, cardiac, survival, cardiopulmonary, bystander, rhythm, shockable, spontaneous, circulation
219	gold, volatility, oil, asset, connectedness, stock, market, hedging, price, portfolio
220	nursing, home, resident, staff, facility, star, incidence, per, mortality, ownership
221	conspiracy, belief, mentality, conspiratorial, theory, narcissism, believing, political, endorsement, thinking
222	drug, discovery, chemical, machine, graph, deep, learning, molecular, computational, prediction
223	allergic, anaphylaxis, peg, allergy, skin, anaphylactic, hypersensitivity, vaccine, polyethylene, glycol
224	breastfeeding, milk, feeding, lactation, exclusive, infant, breast, support, mother, maternal
225	blood, donation, transfusion, donor, supply, donate, plasma, collection, voluntary, demand
226	religious, spiritual, spirituality, religiosity, coping, anxiety, religion, stress, god, fear
227	estate, housing, real, rental, market, price, property, house, residential, rent
228	noise, seismic, acoustic, sound, traffic, annoyance, reduction, anthropogenic, urban, shipping
229	psoriasis, psoriatic, biologic, systemic, exacerbation, pasi, arthritis, treatment, therapy, biological
230	neck, head, cancer, surgery, oral, carcinoma, flap, surgical, oncology, reconstruction
231	audio, cough, speech, sound, acoustic, voice, classification, breathing, deep, vocal
232	retinal, eye, occlusion, ocular, vision, fundus, visual, optic, vein, macular
233	pancreatitis, pancreatic, lipase, acute, amylase, pancreas, abdominal, gastrointestinal, pain, case
234	nepal, nepali, nepalese, government, preparedness, country, migrant, gon, health, road
235	mask, filtration, filter, cloth, respirator, face, fit, fabric, efficiency, surgical
236	delirium, catatonia, agitation, neuropsychiatric, sedation, visitation, delirious, coma, dementia, older
237	radiology, interventional, radiological, radiography, volume, outpatient, shutdown, department, radiologic, practice
238	pharmacology, network, pathway, molecular, enrichment, active, go, encyclopedia, target, mechanism
239	literacy, information, health, digital, knowledge, university, preventive, medium, behavior, level
240	vaccination, anxiety, mental, psychiatric, vaccine, depression, hesitancy, psychological, stress, distress
241	surge, care, hospital, emergency, ambulance, team, york, bed, staff, facility

Topic	Words
242	crime, firearm, gun, theft, violence, burglary, criminal, violent, robbery, police
243	eu, european, union, member, crisis, integration, policy, commission, economic, political
244	vaccine, development, vaccination, safe, regulatory, safety, world, global, immunity, effective
245	smoking, tobacco, former, nicotine, current, status, association, never, severity, risk
246	intention, adoption, acceptance, usefulness, tam, ease, technology, use, expectancy, influence
247	spine, spinal, surgery, chiropractic, elective, surgical, lumbar, practice, cervical, postoperative
248	nab, nabs, neutralization, assay, serum, titer, surrogate, convalescent, antibody, plasma
249	model, hospital, capacity, occupancy, demand, bed, forecast, simulation, number, resource
250	honorarium, advisory, membership, board, bureau, myeloma, leukemia, hematological, lymphoma, myeloid
251	milk, breast, breastfeeding, colostrum, infant, human, pasteurization, passive, maternal, serum
252	wildlife, conservation, specie, zoo, trade, wild, habitat, human, animal, urban
253	pediatric, care, delivery, visit, practice, survey, satisfaction, video, patient, pediatrics
254	adrenal, pituitary, endocrine, insufficiency, apoplexy, gland, hormone, ai, hypopituitarism, thyroid
255	indonesian, government, handling, bali, policy, defense, village, implementation, regional, yang
256	liver, transplant, transplantation, donor, recipient, organ, meld, graft, cirrhosis, list
257	news, journalism, medium, journalistic, newspaper, coverage, content, press, framing, crisis
258	fiscal, debt, monetary, policy, economic, inflation, crisis, growth, deficit, spending
259	chinese, medicine, traditional, western, treatment, decoction, granule, disappearance, formula, efficacy
260	dialysis, antibody, dose, humoral, vaccination, response, vaccine, maintenance, peritoneal, kidney
261	stroke, ischemic, vessel, occlusion, hemorrhagic, cryptogenic, acute, mr, without, discharge
262	cholecystitis, cholecystectomy, biliary, gallstone, cholecystostomy, surgery, pancreatitis, surgical, elective, acute
263	oil, crude, volatility, price, stock, market, energy, uncertainty, index, gasoline
264	social, work, service, profession, professional, practice, interprofessional, article, need, worker
265	dashboard, data, information, visualization, open, interactive, public, map, surveillance, web
266	personality, neuroticism, extraversion, conscientiousness, agreeableness, openness, big, stress, trait, extroversion
267	arthroplasty, knee, hip, elective, tha, surgery, joint, shoulder, revision, waiting
268	tuberculosis, bacillus, vaccination, nonspecific, immunity, protection, vaccine, trained, mycobacterium, immune
269	dog, pet, veterinary, companion, animal, ownership, cat, bite, welfare, attachment
270	iron, metabolism, anemia, serum, hemoglobin, homeostasis, fe, deficiency, chelation, overload
271	household, sar, attack, transmission, secondary, index, close, contact, case, rate
272	mouse, human, animal, ace, lung, pathogenesis, receptor, murine, model, replication

Topic	Words
273	tax, taxation, vat, income, incentive, revenue, taxpayer, policy, economic, fiscal
274	cutaneous, skin, rash, eruption, erythema, hypersensitivity, adverse, vaccine, vaccination, reaction
275	menstrual, cycle, menstruation, bleeding, dysmenorrhea, premenstrual, length, reproductive, menses, stress
276	career, faculty, academic, regret, choice, productivity, research, professional, work, medicine
277	intellectual, disability, life, quality, people, developmental, enterprise, orad, support, id
278	glomerulonephritis, nephropathy, hematuria, renal, kidney, glomerular, vasculitis, biopsy, proteinuria, vaccination
279	cost, spending, hospital, inpatient, per, direct, burden, insured, medical, costing
280	expression, ace, cancer, adenocarcinoma, lung, tumor, gene, prostate, methylation, carcinoma
281	fuzzy, intuitionistic, decision, linguistic, pythagorean, set, aggregation, weighted, rough, method
282	printing, equipment, printed, additive, production, face, fabrication, protective, supply, personal
283	fatigue, long, chalder, persistent, symptom, myalgic, syndrome, chronic, severity, scale
284	vulnerability, index, vulnerable, social, socioeconomic, spatial, housing, census, county, poverty
285	cardiac, surgery, surgical, postoperative, elective, bypass, adult, operative, aortic, underwent
286	simulation, training, session, airway, team, intubation, staff, simulator, equipment, confidence
287	skin, dermatitis, wearing, protective, equipment, facial, acne, dermatological, adverse, mask
288	colchicine, trial, standard, placebo, group, usual, treatment, mortality, mechanical, day
289	temperature, sensor, drone, cloud, body, system, wearable, device, server, mobile
290	wave, second, india, unrestricted, first, third, country, attack, rate, number
291	wave, second, first, mortality, admitted, hospital, third, higher, compare, age
292	perception, risk, behavior, preventive, protective, social, medium, intention, communication, subjective
293	environmental, environment, pollution, water, air, waste, human, soil, noise, global
294	distribution, estimation, maximum, likelihood, function, exponential, simulation, monte, carlo, model
295	patent, waiver, intellectual, property, compulsory, pharmaceutical, innovation, proposal, access, trade
296	china, epidemic, model, reproduction, province, number, quarantine, control, peak, infected
297	informal, poverty, livelihood, sector, income, rural, economic, poor, economy, vulnerable
298	radiotherapy, radiation, oncology, department, treatment, staff, cancer, therapy, coe, patient

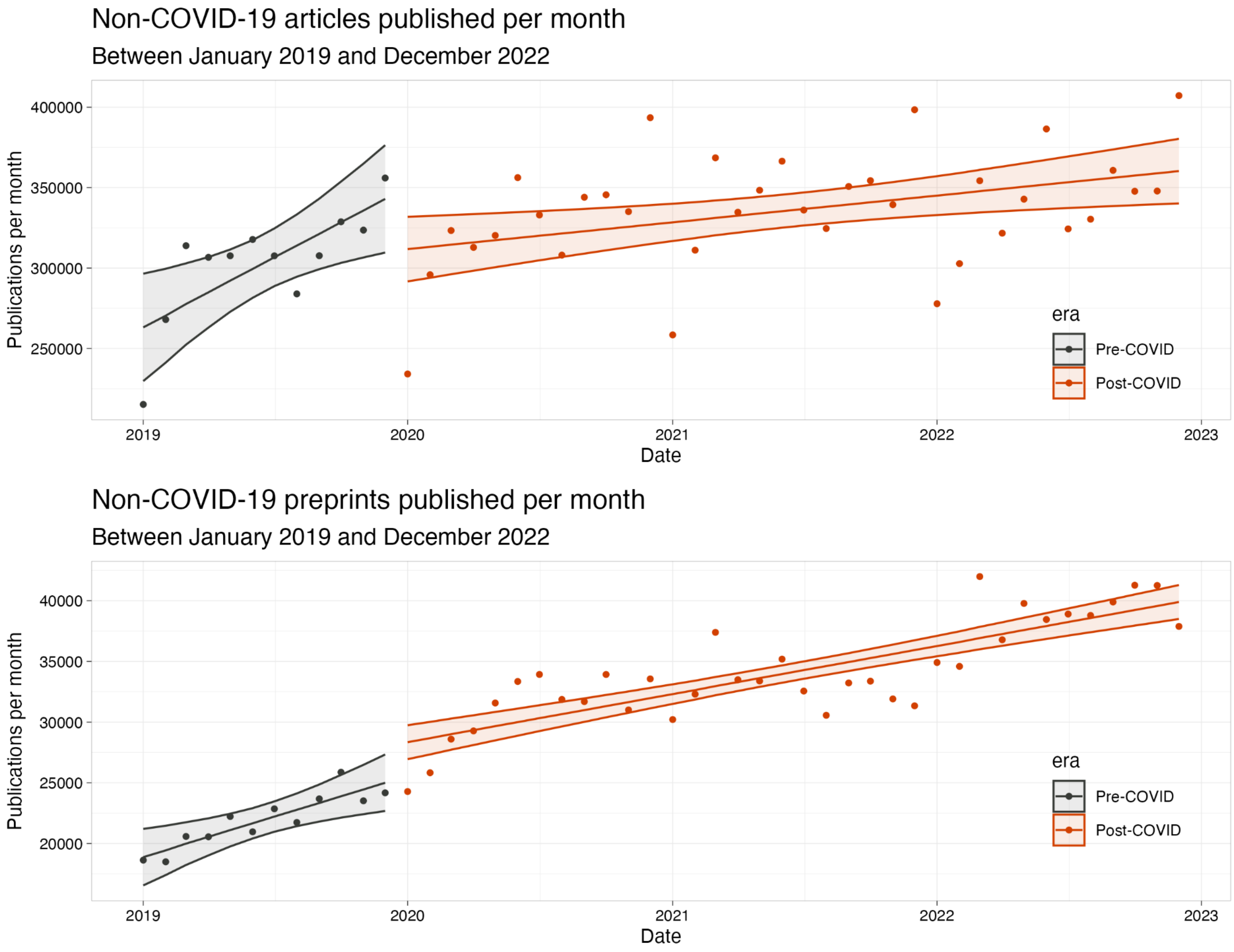

Figure S.1. Publication rates of COVID-19 papers in the pre-pandemic period (2019) and the pandemic period (2020-2022). Regression lines and 95% CIs are shown for regression of articles/month on to time (in months since January 2019) and a binary pre-pandemic/post pandemic variable, plus an interaction effect between these two independent variables. In journal articles, this interaction effect had p-value p=0.03 suggesting a decline in growth rate during the pandemic period. In preprints, the interaction effect had p-value p=0.44, while the p-value for the main effect for the pre/post-pandemic binary variable was p=0.003, with a positive effect, suggesting a boost in preprint publishing and no decline in growth rate.

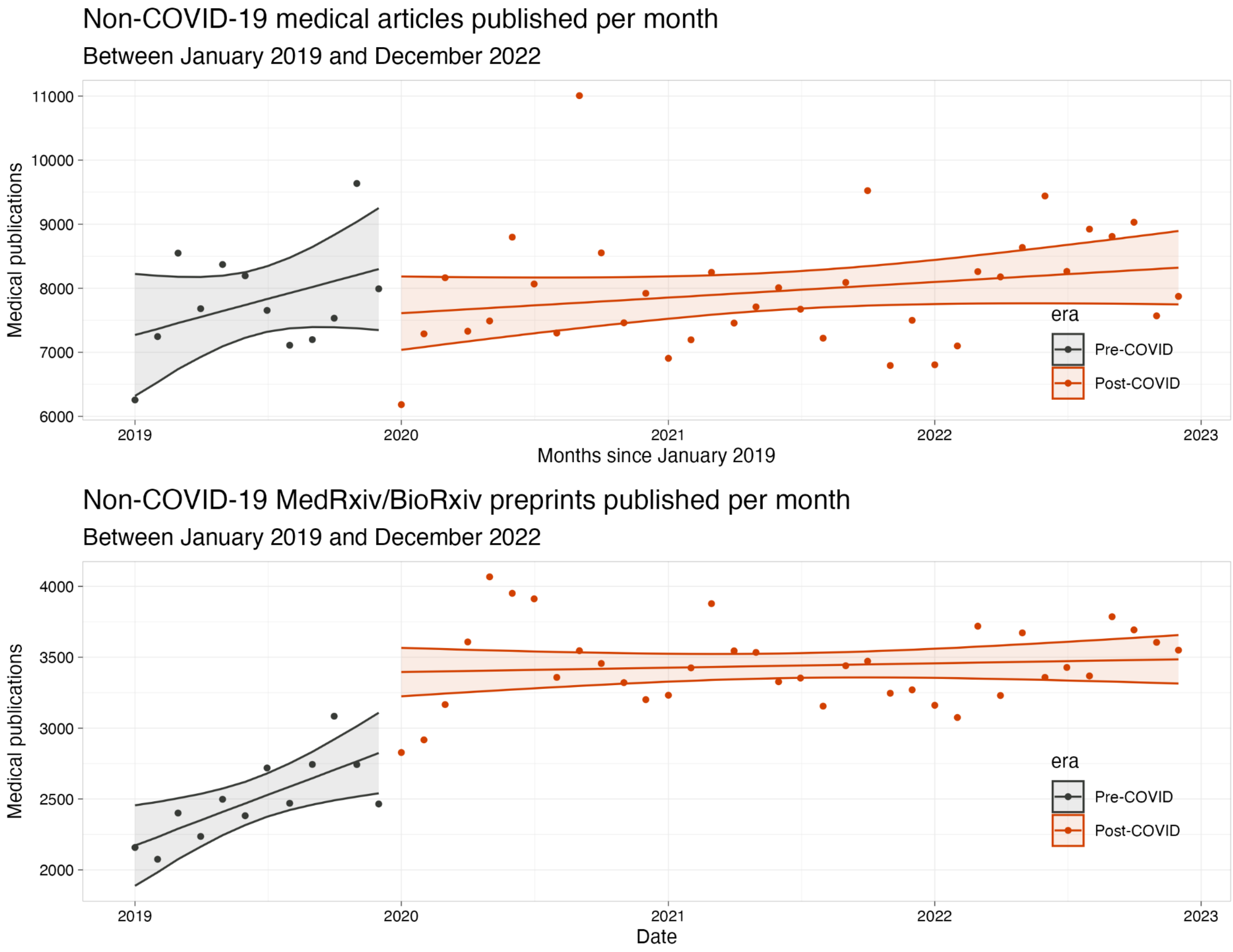

Figure S.2: Publication rates of non-COVID-19 medical papers in the pre-pandemic period (2019) and the pandemic period (2020-2022). Regression lines and 95% CIs are shown for regression of articles/month regressed on time (in months since January 2019) and a binary pre-pandemic/post pandemic variable, plus an interaction effect between these two independent variables. In journal articles, this interaction effect p-value is $p=0.46$, giving no evidence of a change in growth rate during the pandemic period. In preprints, p-values for the main effect for the pre/post-pandemic binary variable, and the interaction effect, were $p<0.00001$ and $p=0.017$ respectively, with a positive effect, suggesting a boost in preprint publishing and a subsequent lower growth rate.

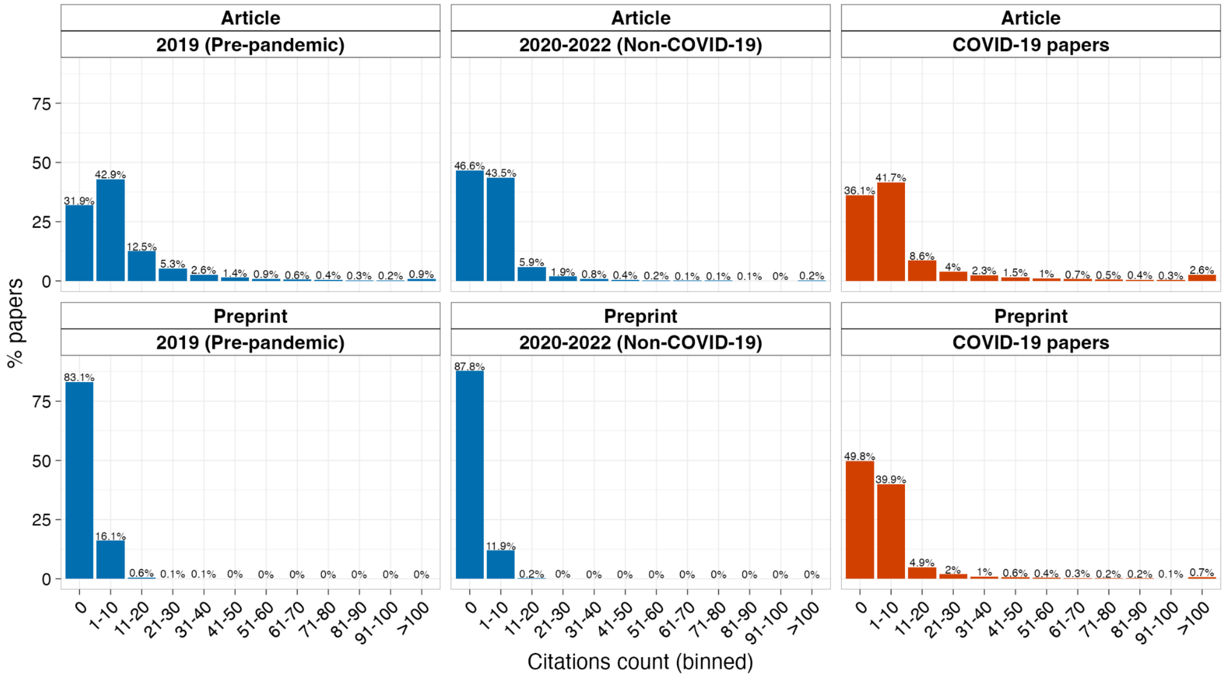

Figure S.3: Distribution of citation counts by article type (preprint/journal article, 2019, 2020-2022[non-COVID] and 2020-2022[COVID]).

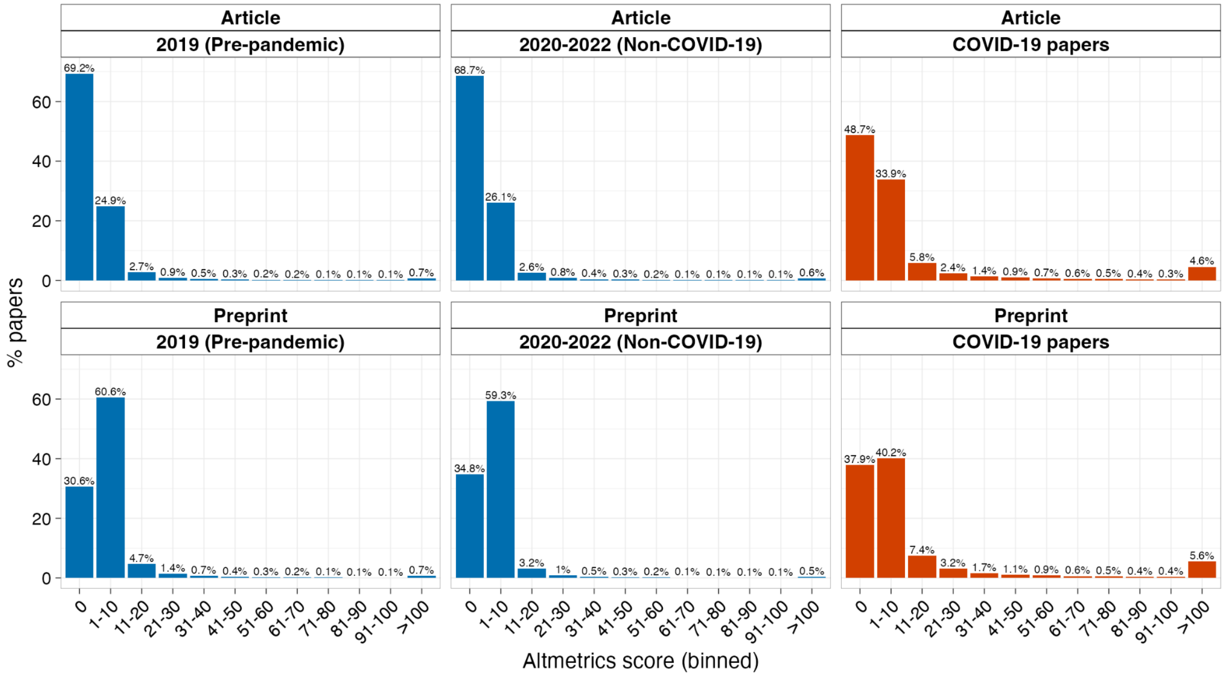

Figure S.4: Distribution of Altmetrics scores by article type (preprint/journal article, 2019, 2020-2022[non-COVID] and 2020-2022[COVID]).

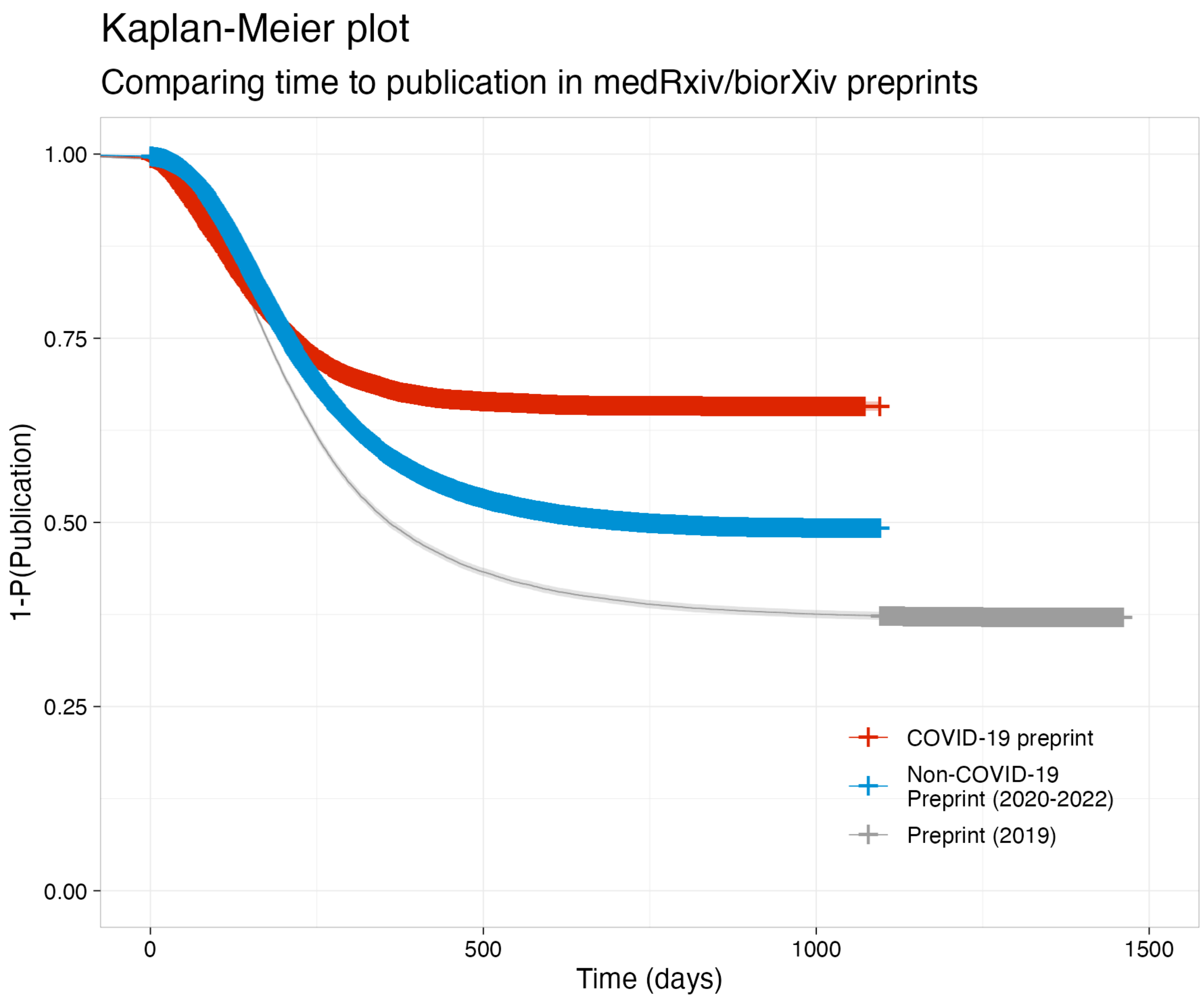

Figure S.5: Kaplan-Meier plots showing time-to-event curves for publication in preprints. COVID-19 preprints had a higher probability of publication in the first~200 days after being preprinted, then a substantially lower probability of publication at all subsequent time points.

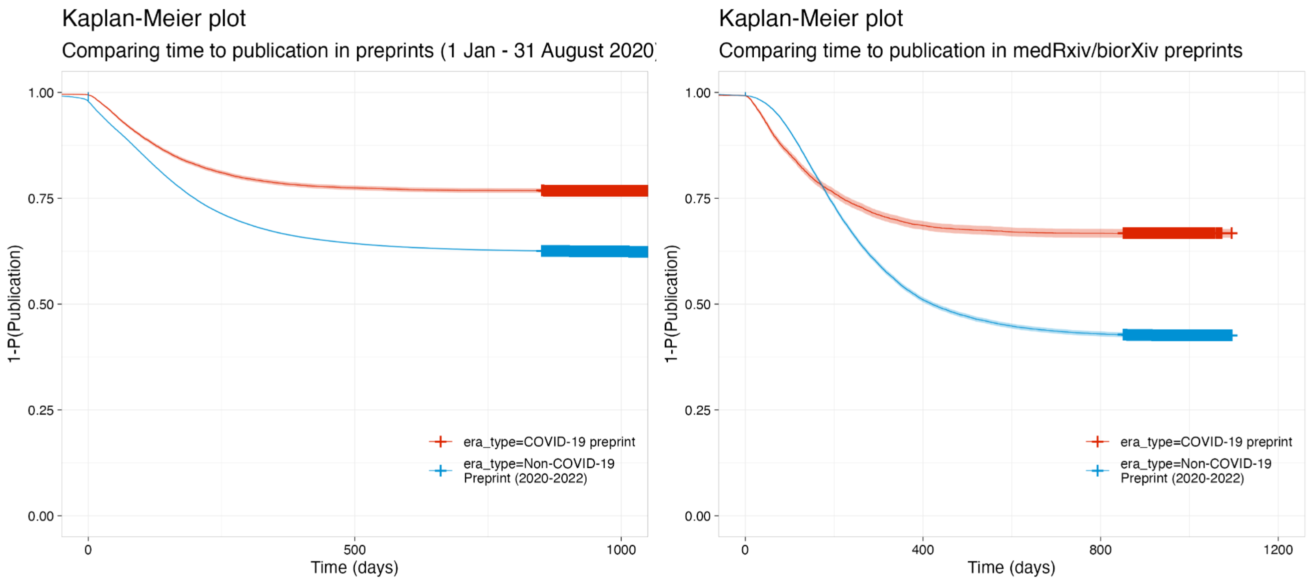

Figure S.6: Kaplan-Meier plots showing time-to-event curves for publication in preprints from the first wave of COVID-19 (to 1 August 2020). Left plot shows time to publication in all preprints; right plot shows time to publication in medRxiv and bioRxiv preprints only. Compared to all other preprints (left plot), COVID-19 preprints had a lower probability of publication at all time points; among bioRxiv and medRxiv preprints only (right plot), COVID-19 preprints had a higher probability of publication in the first ~200 days after being preprinted, then a substantially lower probability of publication at all subsequent time points.

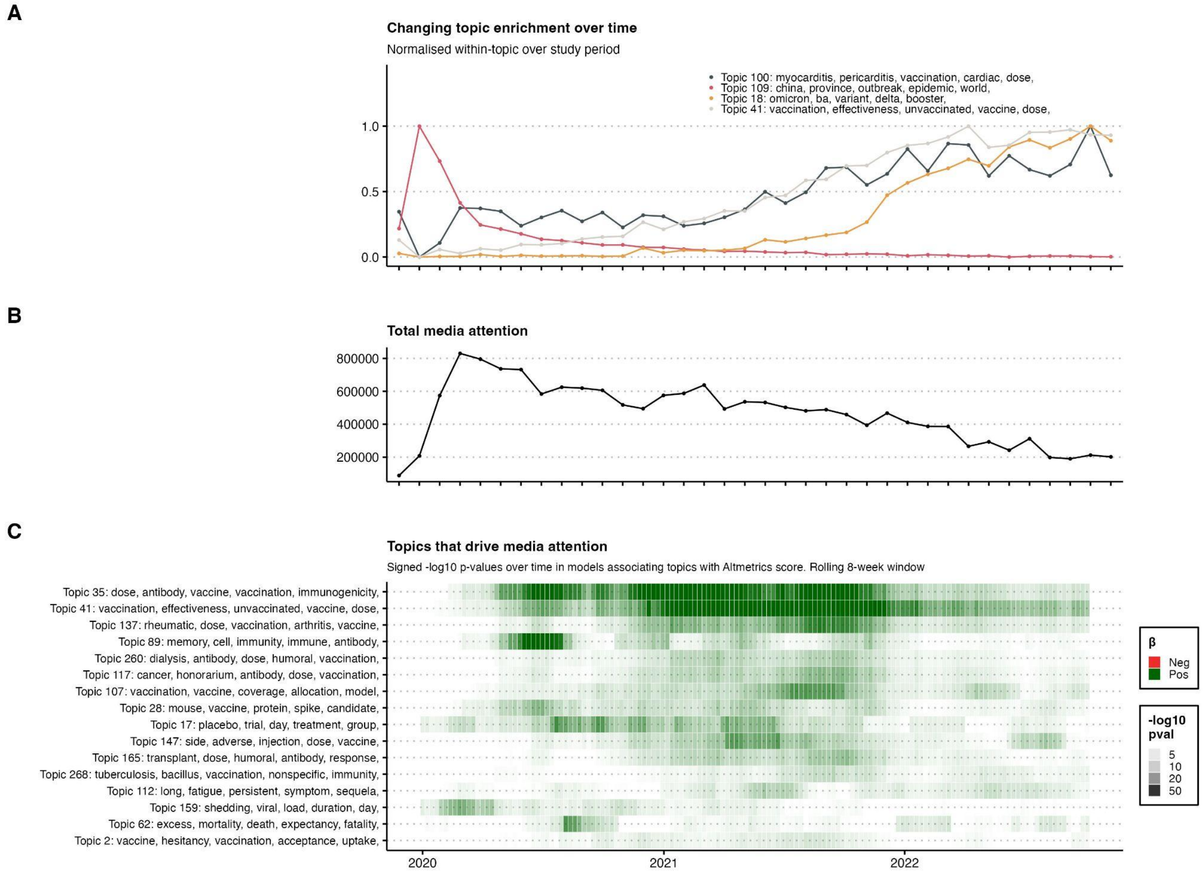

Figure S.7: In journal articles: (A) Line plot showing changes in the relative enrichment of paper abstracts in four topics of particular public health interest, over time. Enrichment is quantified as the mean probability value for each topic across the abstracts published in a given month. (B) Line plot showing total media attention paid to preprints across the study period, shown as a percentage of the peak level. (C) Heatmap showing results of univariable regression of Altmetrics scores onto abstract topic probabilities on a rolling 8-week time window throughout the pandemic period 2020–2022, among preprints only. Colour represents beta values: green = positive betas, red = negative betas and colour darkness represents -log10 p-value value magnitude (darker hue = lower p-value). Topics are ordered by average beta value across the three years, and only topics with at least one period of association (p<0.05) with Altmetrics scores are shown.

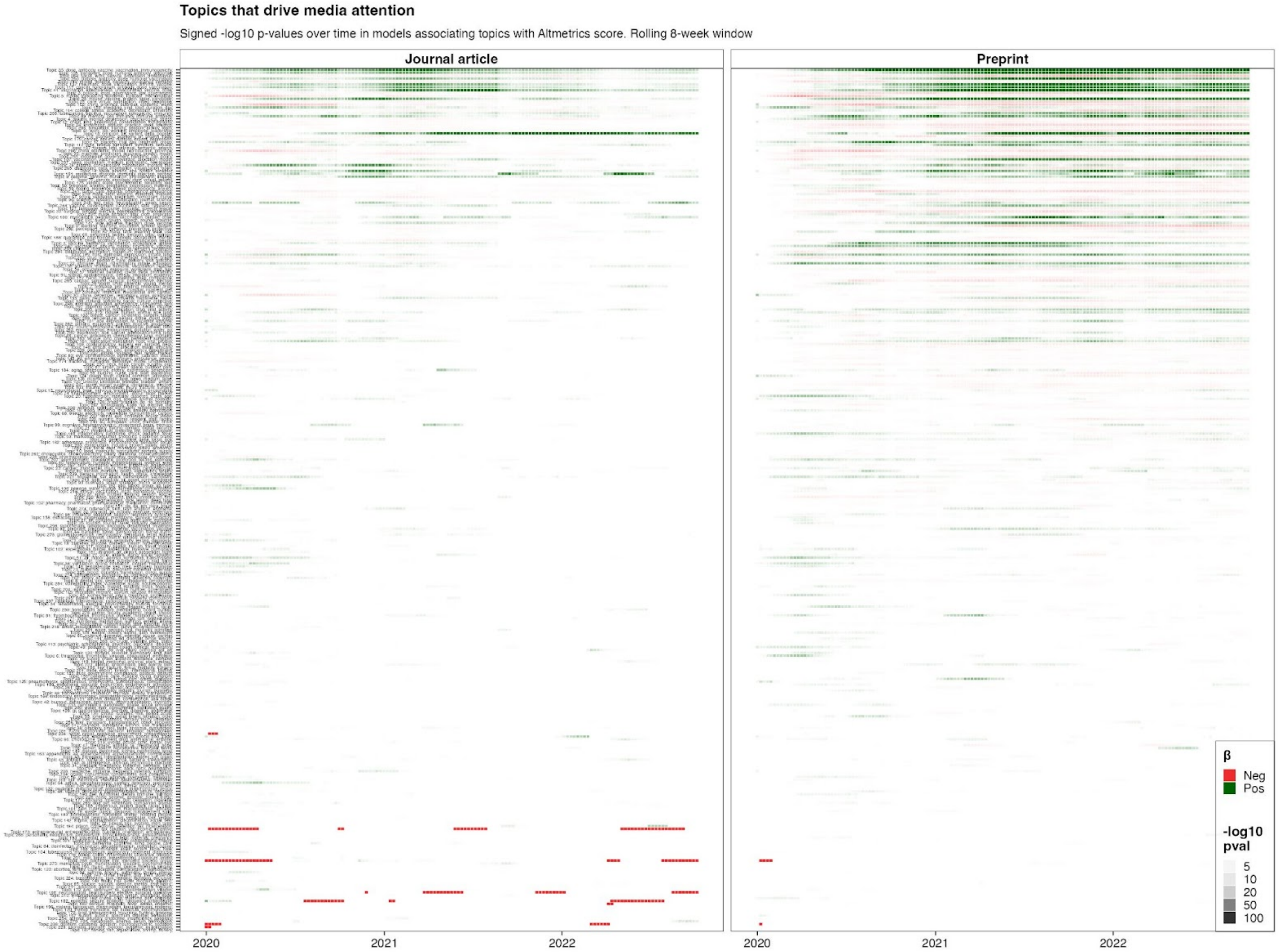

Figure S.8: Comparative heatmap showing results of univariable regression of Altmetrics scores onto abstract topic probabilities on a rolling 8-week time window throughout the pandemic period 2020–2022, among journal articles (left panel) and preprints (right panel). Colour represents beta values: green = positive betas, red = negative betas and colour darkness represents -log10 p-value value magnitude (darker hue = lower p-value). Topics are ordered by average beta value across the three years; all topics are shown.

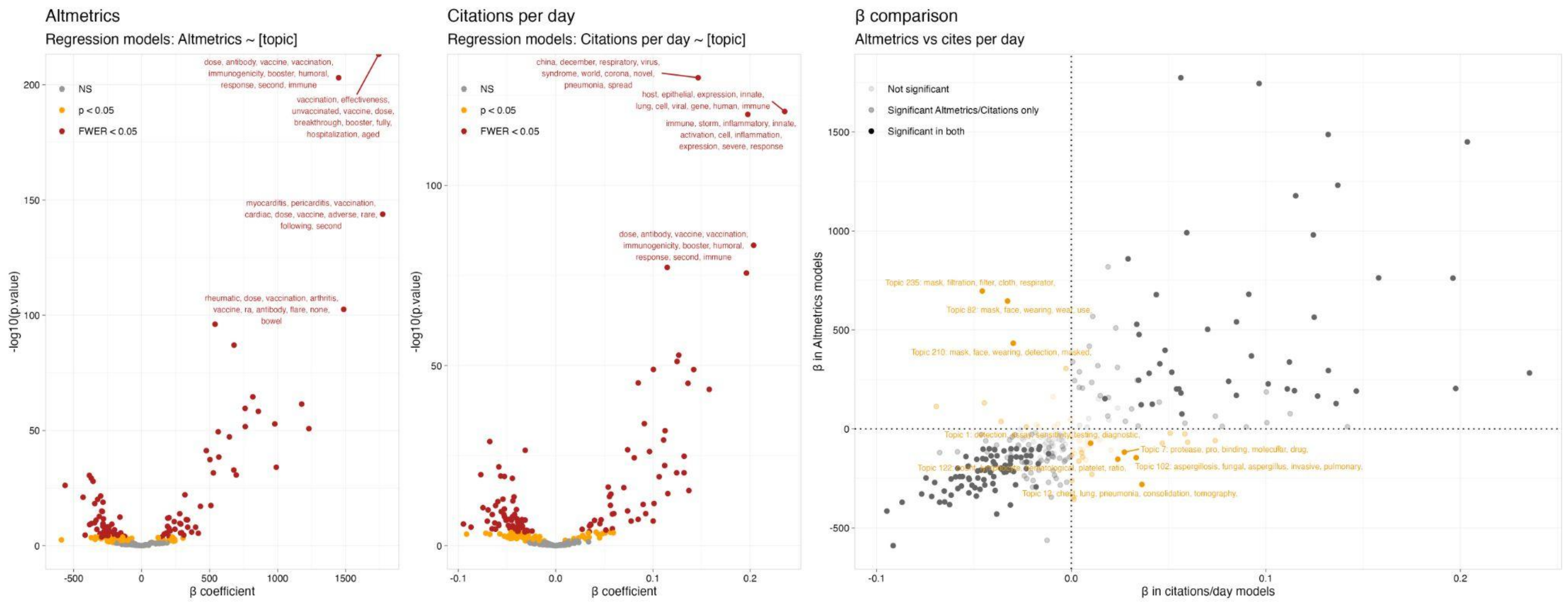

Figure S.9: Panel showing volcano plots of the results of univariable regression of Altmetrics (A) and citation rates (B) onto abstract topic probabilities, and scatter plot comparing beta regression coefficients in topics with associations with citation rates (Y-axis) and Altmetrics (X-axis) (C). Topics for which the regression coefficients were of the same sign for both citation rate and Altmetrics score (N=76, 91.6%) are reported in blue, and those with opposing signs (N=7, 8.4%) are reported in red. *FWER = Family-Wise Error Rate (Bonferroni adjusted p-value)

Supplementary methods

S.1.1 The Dimensions data set

The Dimensions research database is a 'real-time' bibliographic data set that collates multiple different types of research object — peer-reviewed journal articles, preprints, patents, clinical trials, grants and policy documents[1] — from across all fields of science, arts and humanities. Rich metadata are available for each article, including article title and abstract text, author details, publication dates, DOI, citation data, and Altmetrics ID (which permits linking with more extensive Altmetric data through their Application Programming Interface (API)). The database has been shown to have good coverage of scientific articles and to be a viable alternative to non-open-source databases such as Scopus and Web of Science.[2]

S.1.2 Classifying COVID-19 papers

Articles were classified as COVID-19-related if the title text contained any of the following terms:

```
# Original COVID-19 terms
  "2019-nCoV", "COVID-19", "SARS-CoV-2", "HCoV-2019", "hcov", "NCOVID-19",
  "SARS-CoV-2",  "coronavirus disease 2019", "corona virus disease 2019",
  "severe acute respiratory syndrome coronavirus 2",
  "SarsCoV2", "COVID19", "2019-nCov", "COVID 19",
  "severe acute respiratory syndrome corona virus 2",
  "Wuhan coronavirus", "China coronavirus",
  "Wuhan corona virus", "China corona virus",
  "novel coronavirus", "novel corona virus",
  "COVID", "covid", "CoV2", "coronavirus",
  "nCoV-2019", "ncov-2019", "nCoV19", "nCoV",
  "SARS2", "SARS CoV2",

  # Long COVID terms
  "long COVID", "long COVID-19", "long covid", "long-haul COVID",
  "long-haul COVID-19", "post-acute sequelae of SARS-CoV-2 infection",
  "PASC", "post-COVID syndrome", "post-COVID-19 syndrome",
  "chronic COVID syndrome", "long-term effects of COVID-19",

  # Variant terms
```

[1] Hook DW, Porter SJ, Herzog C. Dimensions: Building context for search and evaluation. *Front Res Metr Anal* 2018; **3**. doi:10.3389/frma.2018.00023.

[2] Martín-Martín A, Thelwall M, Orduna-Malea E, Delgado López-Cózar E. Google Scholar, Microsoft Academic, Scopus, Dimensions, Web of Science, and OpenCitations' COCI: a multidisciplinary comparison of coverage via citations. *Scientometrics* 2021; **126**: 871–906.

```
"Alpha variant", "B.1.1.7", "Beta variant", "B.1.351",
"Gamma variant", "P.1", "Delta variant", "B.1.617.2",
"Omicron variant", "B.1.1.529", "SARS-CoV-2 variant",
"COVID-19 variant", "variant of concern", "XBB.1.5",
"BA.4", "BA.5", "XBB", "BA.2.75", "C.37",

# Vaccine-related terms
"Pfizer vaccine", "Moderna vaccine", "AstraZeneca vaccine",
"Johnson & Johnson vaccine"
```

S.1.3 Inferring date of publication

For a minority of papers the full publication date was not available, only the year. In these cases, for the purposes of the publication growth rate analysis, a publication date was randomly assigned from a uniform distribution across the year of publication.

S.1.4 Statistical analysis

Investigating changing publication rates

To investigate changing rates of publishing output, linear regression models were fit to the monthly publication data, with the specification:

$$y = \alpha + \beta X + \epsilon \qquad (1)$$

where y denotes the number of papers published per month and X denotes the corresponding month. To test whether the pandemic era was associated with a change in the rate of publication of non-COVID-19 research, a further regression model was fit to the monthly publication data including a binary independent variable denoting pre-pandemic (2019) or pandemic era (2020-2022), including an interaction between the independent variables:

$$y = \alpha + \beta 1X1 + \beta 2X2 + \beta 1X1 * \beta 2X2 + \epsilon \qquad (2)$$

where y denotes papers per month, X1 months and X2 represents a binary variable indicating the pandemic era (ie post 1 January 2020 y/n).

Altmetrics–citations correlation analysis

We investigated the relationship between scientific attention – as measured by citation count, field citation ratio, journal impact factor and journal citation indicator – and media attention – as measured by the Altmetrics score,

which aggregates all online attention from news and social media – for journal articles. For preprints, we additionally measured scientific attention by whether or not the article had subsequently been published.

S.1.5 Topic modelling

Topic modelling is a method for making large corpuses of text computationally tractable by revealing underlying structure, and it has been shown to be effective for conducting exploratory literature reviews at scale. We applied topic modelling to the abstracts of all COVID 19 papers in the data set of the COVID-19 papers using Bertopic as implemented in the Python package Bertopic.[3]

To prepare the data set, the abstract text corpus was cleaned: papers without abstracts or with incomplete abstracts were excluded altogether; html and xml artefacts, 'stop words' (words that do not add significant meaning to a sentence) and common conjunctions (eg the, and, with) were removed. The text was tokenised (broken down into smaller units, usually individual words) and lemmatized (words are reduced to their roots – eg 'improve' and 'improved' both become 'improve').

The SentenceTransformer model 'all-MiniLM-L6-v2'[4] Uniform Manifold Approximation and Projection (UMAP)[5] was applied to reduce the dimensionality of the vector data. Finally, HDBSCAN[6] (Hierarchical Density-Based Spatial Clustering of Applications with Noise), was used to cluster the documents. HDBSCAN transforms the feature space using the 'mutual reachability distance', and clusters the documents by identifying regions of high density separated by regions of lower density. Finally, topic probabilities were calculated using the approximate_distribution() function to create a document-topic probability matrix, and a set of representative words was assigned to each topic using c-TF-IDF (class-based term frequency–inverse document frequency) scores.

S.1.6 Associating topic enrichment with citations and Altmetrics

To investigate the relationship between article topics and (i) citation rates and (ii) media attention, univariable linear regression models were constructed with y either as citation rate (total citations / days since publication) or Altmetric score and X as the topic probability value. All topics were modelled in this way for each outcome,

---

[3] Grootendorst M. BERTopic: Neural topic modeling with a class-based TF-IDF procedure. arXiv [cs.CL]. 2022.http://arxiv.org/abs/2203.05794.
[4] sentence-transformers/all-MiniLM-L6-v2 · Hugging Face. https://huggingface.co/sentence-transformers/all-MiniLM-L6-v2 (accessed 25 Nov2024).
[5] McInnes L, Healy J, Melville J. UMAP: Uniform Manifold Approximation and Projection for Dimension Reduction. arXiv [stat.ML]. 2018.http://arxiv.org/abs/1802.03426.
[6] McInnes L, Healy J. Accelerated hierarchical density clustering. arXiv [stat.ML]. 2017.http://arxiv.org/abs/1705.07321.

and the results visualised as volcano plots. Regression coefficients were compared across outcomes to see whether the same associations existed for citations as for Altmetrics, and to identify topics that had different effects for the two outcomes. To examine how drivers of attention evolved over the course of the pandemic period, this association analysis was repeated in rolling time windows of 8 weeks between 1 January 2020 and 31 December 2022. For simplicity, attention was assumed to accrue to the paper without lag, in keeping with existing research showing rapid accumulation velocity for Altmetrics.[7]

S.1.7 Linking preprints to subsequent peer-reviewed publications

Several mechanisms exist to detect subsequent publications. First, Crossref monitors preprints and journal articles and, when it detects an article that was previously preprinted, the preprint server is notified. Second, some of the larger preprint servers (eg medRxiv and bioRxiv) monitor their own preprints for subsequent publication and link to the journal article when the preprint authors confirm a link. Finally, authors may notify the preprint server when a preprint has been published in a journal and ask for it to be linked. Approximately 79% of preprints are accurately linked to subsequent publications using these methods.[8]

[7] Fang, Z., Costas, R. Studying the accumulation velocity of altmetric data tracked by Altmetric.com. *Scientometrics* **123**, 1077–1101 (2020). https://doi.org/10.1007/s11192-020-03405-9

[8] Cabanac G, Oikonomidi T, Boutron I. Day-to-day discovery of preprint-publication links. *Scientometrics* 2021; **126**: 5285–5304.
